# Recent Advances in Cartesian-Grid DFT in Atoms and Molecules

**DOI:** 10.3389/fchem.2022.926916

**Published:** 2022-07-22

**Authors:** Sangita Majumdar, Amlan K. Roy

**Affiliations:** Department of Chemical Sciences, Indian Institute of Science Education and Research (IISER) Kolkata, Mohanpur, India

**Keywords:** density functional theory, Cartesian grid, electric response properties, exchange-correlation functional, exact exchange energy, range-separated hybrid functional, optimal tuning, excitation energies

## Abstract

In the past several decades, *density functional theory* (DFT) has evolved as a leading player across a dazzling variety of fields, from organic chemistry to condensed matter physics. The simple conceptual framework and computational elegance are the underlying driver for this. This article reviews some of the recent developments that have taken place in our laboratory in the past 5 years. Efforts are made to validate a viable alternative for DFT calculations for small to medium systems through a Cartesian coordinate grid- (CCG-) based pseudopotential Kohn–Sham (KS) DFT framework using LCAO-MO ansatz. In order to legitimize its suitability and efficacy, at first, electric response properties, such as dipole moment (***μ***), static dipole polarizability (***α***), and first hyperpolarizability (***β***), are calculated. Next, we present a *purely numerical* approach in CCG for proficient computation of exact exchange density contribution in certain types of orbital-dependent density functionals. A Fourier convolution theorem combined with a range-separated Coulomb interaction kernel is invoked. This takes motivation from a semi-numerical algorithm, where the rate-deciding factor is the evaluation of electrostatic potential. Its success further leads to a systematic self-consistent approach from first principles, which is desirable in the development of optimally tuned range-separated hybrid and hyper functionals. Next, we discuss a simple, alternative time-independent DFT procedure, for computation of single-particle excitation energies, by means of “adiabatic connection theorem” and virial theorem. Optical gaps in organic chromophores, dyes, linear/non-linear PAHs, and charge transfer complexes are faithfully reproduced. In short, CCG-DFT is shown to be a successful route for various practical applications in electronic systems.

## 1 Introduction

“The general theory of quantum mechanics is now almost complete⋯ The underlying physical laws necessary for the mathematical theory of a large part of physics and the whole of chemistry are thus completely known, and the difficulty is only that the exact application of these laws leads to equations much too complicated to be soluble. It therefore becomes desirable that approximate practical methods of applying quantum mechanics should be developed, which can lead to an explanation of the main features of complex atomic systems without too much computation” (Dirac, 1929). This famous and enlightening announcement in 1929 made physicists and chemists rise to the challenge and develop the theoretical framework needed to calculate wave functions and properties of atoms, molecules, and solids. Throughout the course of the past few decades, *ab initio* quantum mechanical methods have been utilized for elucidation of electronic structure, properties, and dynamics of many-electron systems. With the rapid progress of computational algorithms, in association with modern computer architecture and resources, some of the recent advancements have been extended to widely explored fields such as materials science, nanoscience, and biological science. The electronic Schrödinger equation (SE) of such an N-electron system is, in principle, a many-body problem having space, spin, and time variables, expressed as

$$H\psi_{i}\left(x_{1},x_{2},\ldots ,x_{N}\right)=E_{i}\psi_{i}\left(x_{1},x_{2},\ldots ,x_{N}\right),\;x=\left\{r,\sigma \right\},$$ (1)

where **H** is the many-electron Hamiltonian operator consisting of *M* nuclei and *N* electrons, *ψ*_*i*_ is the *N*-electron anti-symmetric wave function corresponding to *i*th eigenstate of **H**, and E_*i*_ is its energy. However, to pursue it practically, one has to go through multiple challenges caused due to the size of many-body SE. The exact analytical solution is very hard to obtain in most cases, leaving aside a few well-known model systems. The quantum mechanical solvability of *N*-electron systems is exhausted by hydrogen and helium atom. That is why the question of how to deal with systems containing thousands of electrons and hundreds of nuclei has attained utmost relevance. The straightforward application of SE, either in its numerical, variational, or perturbation theory versions, is nowadays out of the reach of even the most advanced supercomputers. It is for this reason that alternative ways of handling such problems have been vigorously pursued during the last few decades. In the past few years, significant strides have been made in these aforementioned areas.

In today’s computational chemistry, it is desirable to achieve the energy of a chemical reaction within the bounds of chemical accuracy ( < 1 kcal mol^−1^). The primary challenge is to reduce the computational cost without much compromise on accuracy for increasingly larger and complicated systems. Now, starting from less accurate, reliable empirical or semi-empirical schemes to modern *ab initio* methods, all fall under the roof of the current computational chemistry repertoire. Two fundamentally different routes, based on wave function approaches and functional theories, have gained popularity and credence so far.

The usual quantum-mechanical approach to SE can be summarized as follows:

$$v\left(r\right)\overset{\mathrm{SE}}{\Rightarrow }\Psi \left(r_{1},r_{2}\ldots \ldots .,r_{N}\right)\overset{<\Psi |\ldots |\Psi >}{\Rightarrow }\text{observables}.$$ (2)

In other words, one specifies a system by choosing *v*(**r**), plugs it into SE, solves that equation for a wave function Ψ, and then calculates observables by taking the expectation values of operators. Many powerful approximate methods for solving SE have been developed. Thus, starting from the single determinantal uncorrelated Hartree–Fock (HF) to various correlated multi-configurational methods is available. It is well-known that the HF method is still not accurate enough for energy predictions in chemistry; for example, bond energies are significantly underestimated. Thus, post-HF methods, adding numerous other determinants involving excited or “virtual” orbitals, are generally required for a satisfactory prediction. Some notable correlated methods are diagrammatic perturbation theory (based on Feynman diagrams and Green’s functions), Möller–Plesset perturbation theory (MPn), configuration interaction (CI), coupled-cluster ansatz (comes in many flavors such as CCSD, CCSD(T), CCSDT, and CCSDTQ), and multi-reference perturbation theory (such as CASPT2), among others. These methods offer authentic and reliable results but are quite difficult to be implemented computationally for large N mainly because of their unfavorable scaling.

The above-mentioned methods based on an approximation to many-electron wave function were the only possibilities until 1964, the birth year of density functional theory (DFT). In general, the so-called functional theories rely on utilizing the limited information coming from single-particle electron density, *ρ*(**r**), reduced density matrix or Green’s function, and follow variational principle. Amongst them, however, DFT has emerged as the most versatile and outstanding candidate in electronic structure theory. This leads to the central quantity of our present article, namely, the spin-less, single-particle electron density *ρ*(**r**), which is the diagonal element of one-particle density matrix, defined as

$$\rho \left(r\right)=N\int \ldots .\int \Psi \left(r\sigma ,x_{2},\ldots \ldots x_{N}\right)\Psi^{*}\left(r\sigma ,x_{2},\ldots \ldots x_{N}\right)d\sigma dx_{2}\ldots ..dx_{N}.$$ (3)

Walter Kohn noted in his Nobel lecture that “DFT has been most useful for systems of very many electrons where wave function methods encounter and are stopped by the exponential wall” (Kohn, 1999). Many beautiful books and in-depth reviews are available on the subject (Parr and Yang, 1989; Koch and Holthausen, 2001; Martin, 2004; Pugh and Hinchliffe, 2006; Hoffman, 2007; Champagne and Springborg, 2009; Burke, 2012; Roy, 2012; Zangwill, 2015; Yu et al., 2016; Mardirossian and Head-Gordon, 2017; Janesko, 2021).

The first genuine DFT scheme for electronic systems was given as early as in 1927 (Thomas, 1927); it was a model for calculating atomic properties, based purely on *ρ*(**r**). It assumed that electrons form a gas satisfying Fermi statistics, with electron–electron interaction energy determined from classical Coulomb potential. For kinetic energy, they adopted a local density approximation (LDA), where the contribution from a point **r** is determined from kinetic energy of a homogeneous electron gas with this density. The resulting Euler equation is

$$\frac{5}{3}C_{F}\rho^{\frac{2}{3}}\left(r\right)+\int dr^{\prime }\frac{\rho \left(r^{\prime }\right)}{\left|r-r^{\prime }\right|}+v_{\mathrm{ext}}\left(r\right)+\lambda =0,$$ (4)

where $C_{F}=\frac{3}{10}{\left(3\pi^{2}\right)}^{\frac{2}{3}}$ , *v*_*ext*_(**r**) is external potential, and *λ* is the Lagrange multiplier related to the constraint of constant particle number. The Thomas–Fermi (TF) model has severe deficiencies because of poor description of outer region of an atom. The charge density decays as *r* ^−6^ far from nucleus, not exponentially as it should. Moreover, it does not bind neutral atoms or form molecules or solids. Later, Dirac (1930) noted the necessity of incorporating “exchange” phenomena, by recasting HF theory in terms of a “density function,” without reference to a single-determinantal wave function. This function leads to a correction to the energy derived from exchange energy for a homogeneous electron gas of *ρ*(**r**). The modified Thomas–Fermi–Dirac (TFD) energy density functional can be written as

$$E_{\mathrm{TFD}}\left[\rho \left(r\right)\right]=C_{F}\int \rho {\left(r\right)}^{\frac{5}{3}}dr+\int \rho \left(r\right)v_{\mathrm{ext}}\left(r\right)dr-C_{x}\int \rho {\left(r\right)}^{\frac{4}{3}}dr+\frac{1}{2}\iint \frac{\rho \left(r\right)\rho \left(r^{\prime }\right)}{\left|r-r^{\prime }\right|}.$$ (5)

The overwhelming simplicity of abandoning a complicated many-electron SE for a single equation in terms of *ρ*(**r**) alone is remarkably appealing. However, underlying approximations are rather primitive, making it grossly inadequate for any practical use in quantum chemistry.

In 1964–1965, the Hohenberg–Kohn (HK) (Hohenberg and Kohn, 1964) and Kohn–Sham (KS) (Kohn and Sham, 1965) theorems, the twin pillars of modern DFT, were published. They asked a plain obvious question of whether the information contained in *ρ*(**r**) is sufficient for elucidating a many-electron system completely. In these seminal works, they founded the rigorous theory that finally legitimized the intuitive leaps of other previous works. They first proved that the external potential, *v*_*ext*_(**r**), of a non-degenerate ground state of an *N*-particle system (and hence total energy), is a unique functional of ground-state electron density *ρ*(**r**). This one-to-one mapping, which is the basic preamble of this theorem, can be expressed by the following energy functional:

$$E_{v_{\text{ext}}}\left[\rho \right]=\langle \psi \left[\rho \right]|T+V_{\text{ext}}+V_{\text{ee}}|\psi \left[\rho \right]\rangle =F_{\text{HK}}\left[\rho \right]+\int v_{\text{ext}}\left(r\right)\rho \left(r\right)dr,$$ (6)

where *F*_*HK*_[*ρ*] denotes the universal energy density functional containing kinetic energy and electron–electron interaction terms. Further, the total energy of a given system can be determined variationally by minimizing the functional $E_{v_{\mathrm{ext}}}\left[\rho \left(r\right)\right]$ , subject to the normalization condition, *∫ρ*(**r**) = *N*, as a constant, through the following equation:

$$\frac{\delta F_{\text{HK}}\left[\rho \right]}{\delta \rho \left(r\right)}+v_{\text{ext}}\left(r\right)=\mu .$$ (7)

Moreover, to make sure that a given density is indeed the true ground-state density, now the second HK theorem suggests

$$E_{v_{\text{ext}}}\left[\rho \left(r\right)\right]\geq E_{v_{\text{ext}}}\left[\rho_{0}\left(r\right)\right],$$ (8)

where $E_{v_{\mathrm{ext}}}\left[\rho_{0}\left(r\right)\right]$ corresponds to the ground-state energy of a Hamiltonian with *v*_*ext*_(**r**) as external potential and *ρ*_0_(**r**) is its ground-state density.

The explicit form of *F*_*HK*_(*ρ*) is unknown as yet. Hence, this needs to be evaluated approximately. However, so far, the most successful way to implement DFT is through a method proposed in Kohn and Sham (1965). They introduced the clever concept of a fictitious, non-interacting system built from a set of KS orbitals, such that the major part of kinetic energy can be computed exactly. The remaining fairly small portion is absorbed in the non-classical contribution of electron–electron repulsion, which is also unknown. However, the advantage is that electrons now move in an effective KS single-particle potential. The mapped auxiliary system now yields the same ground-state density as the real interacting system. However, this simplifies the actual calculation tremendously, as it is operationally an independent-particle theory, simpler even than HF. Yet, it delivers, in principle, the exact density the same as ground-state density resulting from a summation of moduli of square of orbitals through

$$\rho_{s}\left(r\right)=\overset{N}{\underset{i}{\sum }}\underset{\sigma }{\sum }{\left|\Psi_{i}\left(r,\sigma \right)\right|}^{2}=\rho_{0}\left(r\right).$$ (9)

Here, *σ* signifies spin, and the exact total energy is expressed as

$$E\left[\rho \right]=\int v_{\mathrm{ext}}\left(r\right)\rho \left(r\right)dr+J\left[\rho \right]+T_{s}\left[\rho \right]+E_{xc}\left[\rho \right],$$ (10)

where

$$E_{xc}\left[\rho \right]=\left(T\left[\rho \right]-T_{s}\left[\rho \right]\right)+\left(E_{ee}\left[\rho \right]-J\left[\rho \right]\right)=T_{c}\left[\rho \right]+E_{nc}\left[\rho \right].$$ (11)

The associated terms have the following meanings: *J*[*ρ*] is the known classical part of *E*_*ee*_[*ρ*], whereas *E*_*xc*_[*ρ*] contains everything unknown, that is, non-classical electrostatic effects of *E*_*ee*_[*ρ*] and the difference between true kinetic energy *T*[*ρ*] and *T*_*s*_[*ρ*]. The latter represents the sum of individual kinetic energies of reference system with same density as real system as $T_{s}=-\frac{1}{2}\sum_{i}^{N}\nabla_{i}^{2}$ and *T*_*c*_[*ρ*] symbolizes the correlation kinetic energy.

Thus, as apparent from above, the beauty of DFT lies in its being exact yet efficient, with a single determinant describing the *ρ*(**r**)—the whole complexity is hidden in one term, the exchange-correlation (XC) functional. Local exchange functionals in KS theory automatically include some static correlation (Cook and Karplus, 1987; Handy and Cohen, 2001). Thus, a single Slater determinant is not necessarily as poor in KS theory as in HF theory, keeping the burden at the same level as HF. Building better and improved XC functionals to treat real correlated systems within KS theory is an active area of research (Peverati and Truhlar, 2014; Dale et al., 2017; Verma et al., 2019; Wang et al., 2019; Verma and Truhlar, 2020), so much so that the story behind the success of DFT, to a large extent, is tantamount to the search for accurate reliable XC functional. The exact form remains elusive; it is necessary to use various density functional approximations (DFAs). The popular DFAs can be hierarchically characterized in the following manner. The simplest XC functionals are of LDA-type (Kohn and Sham, 1965) (containing *ρ* only), residing in the first rung of Jacob’s ladder. They are exact for an infinite uniform electron gas but are highly inaccurate for molecular properties because most real systems have inhomogeneous density distribution. Next, generalized gradient approximation (GGA) functionals (Becke and Dickson, 1988; Perdew et al., 1996a) (with the addition of gradient of electron density, ∇*ρ*) occupy the second rung of the ladder and tend to improve significantly upon LDA. In addition to combining separable exchange and correlation functionals (e.g., PBE, BP86, BLYP, PW91, rev-PBE, and RPBE), it is possible to semi-empirically parameterize GGA XC functionals (HCTH, N12, and GAM) (Boese and Handy, 2001; Peverati and Truhlar, 2012a; Yu et al., 2015). Then, the third rung belongs to meta-GGA (Tao et al., 2003; Zhao and Truhlar, 2008) functionals (addition of kinetic energy density, *τ*). With further inclusion of exact exchange (EEX) energy, one gets the hybrid functionals, occupying the fourth rung of the ladder (Perdew and Schmidt, 2000). We also have functionals that go beyond the fourth rung (including virtual orbitals), thus requiring an even higher computational cost.

Now with these insightful backgrounds, we proceed to investigate some aspects of the realistic solution of the KS equation. Therefore, one has to deal with several mathematically non-trivial integrals that cannot be evaluated analytically and pose a certain degree of difficulty. Without such procedures, one is left with no choice but to calculate numerically. It is well recognized that such a discrete procedure for multi-center integrals in 3D space is difficult. It becomes even more daunting when it is noted that more computation time and a larger grid size are often required to achieve a satisfactory level of chemical accuracy due to the involvement of a prohibitively huge number of operations. Cusps in the density and singular nature of Coulombic potential offer a major challenge when constructing such integration routes. Therefore, to accomplish high-accuracy calculations within a reasonable number of quadrature grids, one needs numerical integrators that are both efficient and sophisticated enough to capture the forms of density at a reasonable level. This opens up a wide range of integrators with varying degrees of performance. Of these, two distinct, well-recognized partitioning schemes have shown a great deal of promise: the Voronoi cellular approach and fuzzy cells Avenue, commonly known as the atom-centered grid (ACG). Currently, the enviable status of DFT is beholden to the ensuing basis-set calculations. As these studies are heavily dominated by ACG, the real-space grid has been invoked for fully numerical, basis-set free DFT methods. Apart from ACG, a few scattered works exist for different grids in literature, for example, an adaptive Cartesian grid with a hierarchical cubature technique, a transformed sparse-tensor product grid, and a Fourier Transform Coulomb method interpolating density from ACG to a more regular grid. This work presents a simple fruitful way to implement DFT within the basis-set framework, utilizing only CCG.

Thus, within the Born–Oppenheimer approximation and Hohenberg–Kohn–Sham framework, an implementation of DFT is offered in CCG. With the aid of a linear combination of Gaussian functions, molecular orbitals (MO) and quantities such as basis functions, *ρ*(**r**), as well as classical Hartree and non-classical XC potentials, are directly calculated on a 3D real CCG. A combination of Fast Fourier Transform (FFT) and inverse FFT is used to calculate the Coulomb potential quite accurately. In order to present the inner core electrons, analytical effective core potentials are used, whereas energy-optimized truncated Gaussian bases are used for valence electrons. The requisite work of a many-electron problem has four different proceeding directions: method development, improvement of respective new and existing theories, successful implementation of them, and finally, application of these theories in various physicochemical problems. All four genres are covered in this review. Section 2 forms the theoretical backbone for works presented throughout, followed by a systematic investigation of static linear response properties for a host of atoms and molecules in Section 3. Within the finite-field (FF) KS framework, several properties such as permanent dipole moment (***μ***), dipole polarizability (***α***), and first hyperpolarizability (***β***) are considered within the InDFT program (Roy et al., 2019) developed in our laboratory. A simple alternative of the FF technique, employing a rational function fit to ***μ*** with respect to the electric field, is also acquired. Next, a purely numerical, efficient computation scheme of HF exchange energy, density, and matrix elements for certain orbital-dependent and range-separated hybrid (RSH) functionals is presented in Section 4. This is inspired by a recently developed algorithm by Liu and Kong (2017), where an accurate evaluation of the electrostatic potential (ESP) integral is the rate-determining step. A combination of the Fourier convolution theorem (FCT) with an RS Coulomb interaction kernel (CIK) is introduced. The latter is efficiently mapped onto a real grid through an easy optimization procedure, leading to a constraint within the RS parameter, allowing a logarithmic scaling with respect to atomic/molecular size. Simultaneously, as an offshoot of this work, in Section 5, a self-consistent systematic optimization procedure, from first principles, is proposed for the development of optimally tuned (OT) RSH functionals through a size-dependency-based ansatz. To this end, a novel self-consistent procedure appears, which engages the characteristic length of a system with the RS parameter. Finally, Section 6 details the successful implementation of Becke’s exciton model, followed by its applications in computing the optical gap in organic chromophores and various properties of charge-transfer complexes. This is an alternative time-independent DFT procedure to compute single-particle excitation energies, which are of particular relevance in photochemistry. Correct evaluation of the correlated singlet-triplet splitting (STS) energy is the key step in this procedure. Non-empirical models from both the “adiabatic connection theorem” and “virial theorem” are introduced for such calculations. The obtained results are compared with theoretical and experimental references as appropriate. Finally, we end with a few comments in Section 7.

## 2 DFT in Cartesian Grid

In this segment, we briefly outline the methodology and the computational aspects. The single-particle KS equation of a many-electron system in presence of pseudopotential can be written as follows (atomic unit employed unless stated otherwise):

$$\left[-\frac{1}{2}\nabla^{2}+v_{\text{ion}}^{pp}\left(r\right)+v_{h}\left[\rho \left(r\right)\right]+v_{xc}\left[\rho \left(r\right)\right]\right]\phi_{i}\left(r\right)=\epsilon_{i}\phi_{i}\left(r\right),$$ (12)

where $v_{\mathrm{ion}}^{\text{pp}}$ designates ionic pseudopotential, expressed as

$$v_{\text{ion}}^{\text{pp}}=\underset{R_{a}}{\sum }v_{\text{ion},a}^{pp}\left(r-R_{a}\right).$$ (13)

In the above equation, $v_{\text{ion},a}^{\text{pp}}$ represents the ion-core pseudopotential associated with atom *a*, situated at **R**_*a*_, whereas $v_{h}\left[\rho \left(r\right)\right]=\int \frac{\rho \left(r^{\prime }\right)}{\left|r-r^{\prime }\right|}dr^{\prime }$ describes usual classical electrostatic (Hartree) potential among valence electrons, and *v*_*xc*_[*ρ*(**r**)], as usual, represents the non-classical part of many-electron Hamiltonian, **H** as $\frac{\delta E_{\text{xc}}\left[\rho \left(r\right)\right]}{\delta \rho \left(r\right)}$ , the functional derivative of XC energy. The single-particle charge density is then given by

$$\rho \left(r\right)=\rho^{\alpha }\left(r\right)+\rho^{\beta }\left(r\right)=\underset{i}{\sum }f_{i}^{\alpha }|\phi_{i}^{\alpha }\left(r\right){|}^{2}+\underset{i}{\sum }f_{i}^{\beta }|\phi_{i}^{\beta }\left(r\right){|}^{2},$$ (14)

where $\left\{\phi_{i}^{\sigma },\sigma =\alpha \;or\;\beta \right\}$ corresponds to a set of *N* occupied orthonormal spin molecular orbitals (MO) and $f_{i}^{\sigma }$ ’s denote occupation numbers of *i*th spin-MO. The integral of this pseudo-valance density offers the total number of valence electrons, *N*_*v*_. The benefits of pseudopotential are twofold. Firstly, as the number of KS orbitals relies exclusively on *N*_*v*_ only, it is particularly favorable for heavy elements. In such cases, where each atom involves several tens of electrons, *N*_*v*_ can be much smaller. Thus, pseudopotential can consider the non-negligible relativistic impacts in heavy elements. Secondly, as pseudo-valance orbitals are typically smoother than core orbitals, much lesser grid points suffice.

For the realistic solution of Eq. 12, the basis-set technique is by far the most convenient and pragmatic one. This is essentially motivated from the success of basis-set related methodologies in conventional wave function (such as HF and post-HF) theory through LCAO-MO ansatz. Consequently, the KS MOs are expanded in terms of *K* appropriately picked, known basis functions {*χ*_*μ*_(**r**); *μ* = 1, 2, 3, …., *K*}, often called atomic orbitals (AO), in a practice homologous to that in the Roothaan-HF method, such as

$$\phi_{i}\left(r\right)=\overset{K}{\underset{\mu =i}{\sum }}C_{\mu i}\chi_{\mu }\left(r\right),\;i=1,2,3,\ldots .K.$$ (15)

The electron density then takes the following expression in this basis

$$\rho \left(r\right)=\overset{N_{v}}{\underset{i=1}{\sum }}\overset{K}{\underset{\mu =1}{\sum }}\overset{K}{\underset{\nu =1}{\sum }}C_{\mu i}C_{\nu i}\chi_{\mu }\left(r\right)\chi_{\nu }\left(r\right).$$ (16)

In principle, one requires a complete basis set (*K* = *∞*) to get exact expansion of MOs, but it is not achievable in reality. Subsequently, appropriate truncation is needed for practical computational purposes; it suffices to work with a finite basis set.

Now embedding Eq. 15 in Eq. 12, multiplying left side of resulting equation with $\chi_{\nu }^{*}\left(r\right)$ , and then integrating over entire space, trailed by some algebraic manipulation gives rise to the following KS matrix equation, in parallel to the HF case:

$$F^{\text{KS}}C=SC\epsilon ,$$ (17)

where **F** and **S** stand for *K* × *K* real, symmetric total KS and overlap matrices, respectively. The eigenvector matrix **C** contains basis-set expansion coefficients **C**_*μi*_ and diagonal matrix ***ϵ*** holds orbital energies *ϵ*_*i*_. It could be promptly solved by standard numerical techniques of linear algebra. Individual components of KS matrix can be expressed as

$$\begin{array}{ccc} F_{\mu \nu }^{\text{KS}} & = & \int \chi_{\mu }\left(r\right)\left[h^{\text{core}}+v_{\text{hxc}}\left(r\right)\right]\chi_{\nu }\left(r\right)dr\\ & = & H_{\mu \nu }^{\text{core}}+\langle \chi_{\mu }\left(r\right)|v_{\text{hxc}}\left(r\right)|\chi_{\nu }\left(r\right)\rangle =H_{\mu \nu }^{\text{core}}+J_{\mu \nu }+V_{\mu \nu }^{\text{xc}}\\ v_{\text{hxc}}\left(r\right) & = & v_{\text{h}}\left(r\right)+v_{\text{x}}\left(r\right)+v_{\text{c}}\left(r\right), \end{array}$$ (18)

where $H_{\mu \nu }^{\text{core}}$ includes the core bare-nucleus Hamiltonian matrix element comprising of kinetic energy and nuclear-electron attraction, representing 1e^−^ contributions. These can be evaluated analytically with the assistance of well-established recursion relations (Obara and Saika, 1986) for Gaussian type orbital (GTO) bases, which is employed here (see later). The second term *v*_hxc_(**r**) contains all 2e^−^ interactions including classical Coulomb repulsion and non-classical XC potential. *J*_*μν*_ signifies matrix element of *v*_h_(**r**), whereas the remaining term, $V_{\mu \nu }^{\text{xc}}$ supplies XC contribution into 2e^−^ matrix element, whose development remains one of the fundamental steps in the entire KS-DFT process. In absence of any analytical method, the latter can be either computed numerically or fitted by an auxiliary set of Gaussian functions (Sambe and Felton, 1975; Dunlap et al., 1979a; Dunlap et al., 1979b). The current procedure does not engage any fitting. For gradient-corrected functionals, non-local XC contribution of KS matrix is implemented by a finite-orbital basis expansion, without requiring an evaluation of the density Hessians. Thus, in such cases, XC contribution is written in a convenient working form, given in (Pople et al., 1992)

$$F_{\mu \nu }^{\text{xc}\alpha }=\int \left[\frac{\partial f}{\partial \rho_{\alpha }}\chi_{\mu }\chi_{\nu }+\left(2\frac{\partial f}{\partial \gamma_{\alpha \alpha }}\nabla \rho_{\alpha }+\frac{\partial f}{\partial \gamma_{\alpha \beta }}\nabla \rho_{\beta }\right)\cdot \nabla \left(\chi_{\mu }\chi_{\nu }\right)\right]dr,$$ (19)

where *γ*_*αα*_ = |∇*ρ*_*α*_|^2^, *γ*_*αβ*_ = ∇ *ρ*_*α*_ ⋅∇ *ρ*_*β*_, *γ*_*ββ*_ = |∇*ρ*_*β*_|^2^, and *f* is a function only of local quantities *ρ*_*α*_, *ρ*_*β*_, and their gradients.

To continue further, all relevant quantities, such as basis function, electron density, MO, and all 2e^−^ potentials are straightforwardly set up on a real 3D CCG:

$$r_{i}=r_{0}+\left(i-1\right)h_{r},\;i=1,2,3,\ldots .,N_{r};\;for\;r\in \left\{x,y,z\right\},$$ (20)

where *h*_*r*_, *N*_*r*_ imply grid spacing and total number of grid points, respectively, $\left(r_{0}=-\frac{N_{r}h_{r}}{2}\right)$ . As necessary, the single-particle density in the active grid can be expressed as

$$\rho \left(r_{g}\right)=\underset{\mu \nu }{\sum }P_{\mu \nu }\chi_{\mu }\left(r_{g}\right)\chi_{\nu }\left(r_{g}\right),$$ (21)

where **r**_*g*_ is the *g*th grid point in the simulation box. Similarly, any multi-centre integration involved in KS-DFT, such as classical Hartree energy and XC energy, in principle, can be directly set up in 3D real-space grid utilizing small 3D unit volume:

$$I=\int F\left(r\right)dr\approx \underset{i}{\sum }F\left(r_{i}\right)h_{x}h_{y}h_{z}.$$ (22)

The 2e^−^ matrix elements can be acquired by direct numerical integration in CCG:

$$\langle \chi_{\mu }\left(r\right)|v_{\mathrm{hxc}}\left(r\right)|\chi_{\nu }\left(r\right)\rangle \approx h_{x}h_{y}h_{z}\underset{\text{grid}}{\sum }\chi_{\mu }\left(r\right)v_{\mathrm{hxc}}\left(r\right)\chi_{\nu }\left(r\right).$$ (23)

The pragmatic implementation of Eq. 22 is a lot less involved than that in ACG. We note that the presence of a cusp in density and singularity in Coulomb potential would create some computational burden if *F*(**r**) is not smooth enough throughout the simulation box.

A pressing issue in the grid-based approach comprises an accurate estimation of *v*_*h*_(**r**). For finite systems, the simplest and crudest route for computing this is through direct numerical integration on the grid. For smaller systems, this is a feasible option; in any remaining cases, it is generally tedious and cumbersome. However, the most rewarding and widespread approach is through a solution of the corresponding Poisson equation:

$$\nabla^{2}v_{h}\left(r\right)=-4\pi \rho \left(r\right).$$ (24)

The standard method for tackling this is by conjugate gradients (Saad, 2003) or multi-grid solvers (Brandt, 1977). As another option, a conventional FCT was originally suggested by Martyna and Tuckerman (1999), Mináry et al. (2002), Skylaris et al. (2002) and adapted in the context of molecular modeling (Hine et al., 2011; Chang et al., 2012). To give a layout of this theorem, let us start with two functions *f*(**r**) and *F*(**k**) in **r** and **k** spaces, which are Fourier transforms (FT) (denoted by F ) of one another as follows:

$$\begin{array}{ccc} f\left(r\right) & = & F\left(k\right)=\int_{-\infty }^{\infty }F\left(k\right)e^{2\pi irk}dk\\ F\left(k\right) & = & F^{-1}\left(r\right)=\int_{-\infty }^{\infty }f\left(r\right)e^{-2\pi irk}dr. \end{array}$$ (25)

With two functions *f*(**r**), *g*(**r**), one can frame the convolution, characterized by

$$f⋆g=\int_{-\infty }^{\infty }f\left(r^{\prime }\right)g\left(r-r^{\prime }\right)dr^{\prime }.$$ (26)

The FCT directs that FT of convolution is just the product of individual FTs:

$$F\left(f⋆g\right)=F\left(k\right).G\left(k\right),\;\;\;\;\;\;\;\;f⋆g=F^{-1}\left(F\left(k\right).G\left(k\right)\right).$$ (27)

The above theorem can be utilized to construct *v*_*h*_(**r**) efficiently as follows:

$$v_{h}\left(r\right)=FFT^{-1}\left\{v_{h}^{c}\left(k\right)\rho \left(k\right)\right\}\;and\;\rho \left(k\right)=FFT\left\{\rho \left(r\right)\right\},$$ (28)

where $v_{h}^{c}\left(k\right)$ and *ρ*(**k**) represent Fourier integrals of the CIK and density. The quantity *ρ*(**k**) can be handily calculated using discrete FT of its real-space value. Accordingly, our essential worry here lies in the calculation of CIK, which has a singularity in real space. For this, we use an Ewald summation-type approach (Chang et al., 2012), expanding the Hartree kernel into long-range (LR) and short-range (SR) parts:

$$v_{h}^{c}\left(r\right)=\frac{erf\left(\zeta r\right)}{r}+\frac{erfc\left(\zeta r\right)}{r}\equiv v_{h_{\text{lr}}}^{c}\left(r\right)+v_{h_{\text{sr}}}^{c}\left(r\right),$$ (29)

where erf(x) and erfc(x) denote error function and its complementary function, respectively. The FT of SR part can be dealt with analytically, whereas the LR segment necessitates to be computed directly from FFT of real-space values. A convergence parameter *ζ* is utilized to adjust the range of $v_{h_{\text{sr}}}^{c}\left(r\right)$ , so that the error is minimized. Following the conjecture of Martyna and Tuckerman (1999), here we employ *ζ* × *L* = 7 (*L* indicates smallest side of the box), which produces quite precise outcomes (Ghosal et al., 2016; Ghosal et al., 2018; Ghosal et al., 2019). A few other courses that evaluate LR part proficiently are fast multi-pole (Beatson and Greengard, 1997), multi-level summation (Skeel et al., 2002), and fast Fourier-Poisson (York and Yang, 1994) method, among others.

Next, we follow a simple grid-optimization technique as follows:

$${\left\{N_{x},N_{y},N_{z}\right\}}_{i}\;\Leftrightarrow \;\rho_{i}^{\text{SCF}}\;\Leftrightarrow \;E_{\text{tot},i}^{\text{SCF}},$$ (30)

for a fixed grid spacing *h*_*r*_. Here, “*i*” denotes the *i*th combination of *N*_*x*_, *N*_*y*_, *N*_*z*_ for the active box. With this grid parameter, the corresponding self-consistent field (SCF) density and total energy can be labelled as $\rho_{i}^{\text{SCF}}$ and $E_{\text{tot},i}^{\text{SCF}}$ , respectively. Now, one can systematically increase the value of {*N*_*x*_, *N*_*y*_, *N*_*z*_} from an appropriate starting point and eventually reach the optimal value of {*N*_*x*_, *N*_*y*_, *N*_*z*_} such that

$${\left\{N_{x},N_{y},N_{z}\right\}}_{i}={\left\{N_{x},N_{y},N_{z}\right\}}_{\text{opt}},\;when\;\;\;\;\;\;\;\;\;\;\Delta E=\left(E_{\text{tot},i}^{\text{SCF}}-E_{\text{tot},i-1}^{\text{SCF}}\right)<thresh,$$ (31)

where *thresh* is the grid accuracy for total energy convergence, that is, the energy difference between two successive calculations with different grid parameters. A detailed demonstration of this simple optimization strategy has been well documented (Ghosal et al., 2016; Ghosal et al., 2018; Mandal et al., 2019).

For practical, useful electronic structure calculation, it is of foremost significance to choose suitable functions that imitate KS MOs as precisely as possible. Numerical accuracy of KS-DFT is very delicate to the choice and design of a basis set for a particular problem, as an incomplete basis set inducts certain restrictions on the relaxation of density through KS orbitals. A sizeable number of elegant, flexible, versatile basis sets have been proposed from various perspectives, of which GTOs stand out as our most appealing choice. Moreover, for a practical purpose, rather than involving individual GTOs as the basis, it is customary to utilize a fixed linear combination of GTOs, called contracted GTOs. It is cordial in terms of ease and efficiency of computation of essential integrals. While man attractive choices exist for full calculations, which contain higher angular momentum orbitals, the option is much restricted for pseudopotential approximations. We have employed the following effective core potential basis sets: SBKJC (Stevens et al., 1984) for species containing Group-II elements, LANL2DZ (Wadt and Hay, 1985) for Group-III or higher group elements, and Labello–Ferreira–Kurtz (LFK) basis as proposed in Labello et al. (2005), based in the light of a method to incorporate diffuse and polarization functions in familiar Sadlej basis set (Sadlej, 1992). These are adopted from EMSL Basis Set Library (Feller, 1996). All 1e^−^ integrals are generated by standard recursion relations (Obara and Saika, 1986) utilizing GTOs as primitive basis functions. The norm-conserving pseudopotential matrix elements on a contracted basis are imported from the GAMESS (Schmidt et al., 1993) program package. The discrete Fourier transforms are incorporated from the FFTW3 package (Frigo and Johnson, 2005). The above features are implemented in the InDFT (Roy et al., 2019) program developed in our laboratory over the years, which has been employed in several practical applications in a series of articles (Roy, 2008a; Roy, 2008b; Roy, 2010a; Roy, 2010b; Roy, 2011; Ghosal et al., 2016; Ghosal et al., 2018; Ghosal et al., 2019; Mandal et al., 2019; Ghosal et al., 2021; Roy et al., 2021; Ghosal and Roy, 2022a; Ghosal and Roy, 2022b; Roy et al., 2022). At this point, this includes some of the frequently used, prominent XC functionals mentioned at relevant places in the article. The following sections summarize some of the applications of InDFT that have taken place in our laboratory in almost the last 5 years.

## 3 Electric Response Properties

A salient feature of atoms, molecules, and clusters is the electric dipole polarizability; in other words, their ability to respond to an external electric field (George, 2006; Champagne and Springborg, 2009). An accurate description of this has a prominent role in exploring various interesting phenomena of field-matter interaction and inter-particle collision, such as Rayleigh and Raman scattering, second-order Stark effect, and electron detachment process (El Ghazaly et al., 2005). The linear and nonlinear electric properties, such as ***μ***, ***α***, ***β*** are highly relevant in many applications, for example, the development of nonlinear optical materials, structural identification of atomic clusters, Raman and infrared spectroscopy, and separations of molecular isomers. Note that these symbols have also been used for basis set indices. However, there should be no confusion, as their meanings would be apparent from the context of their usage. From a technological point of view, it is interesting to synthesize and design novel optical materials and molecular assemblies with large non-linear optical coefficients (Kümmel and Kronik, 2006). Several distinct theoretical routes were put forward in the literature to obtain these properties within the KS-DFT rubric. Some of the noteworthy ones are the coupled-perturbed Kohn–Sham (CPKS) method (Fournier, 1990; Colwell et al., 1993), linear-response time-dependent DFT (Jansik et al., 2005; Helgaker et al., 2012), perturbative sum-over state expression over all dipole-allowed electronic transitions (Orr and Ward, 1971; Bishop, 1994), the numerical method using the Sternheimer approach (Talman, 2012), auxiliary DFT (Flores-Moreno and Köster, 2008; Carmon-Espíndola et al., 2012), non-iterative CPKS (Shophy et al., 2008) and the fully numerical FF method (Bishop and Pipin, 1987; Maroulis and Thakkar, 1988). The least expensive method, from a computational standpoint, is FF. This approach does not require any analytical derivatives or information about the excited state; implementation is also quite favorable because only the one-body Hamiltonian is perturbed by the applied field (Kurtz et al., 1990). These are the reasons for its success and popularity over other methods (Bulat et al., 2005; de Wergifosse et al., 2014; Wouters et al., 2016).

### 3.1 FF KS Method

The response of a many-electron system can be represented by expanding field-dependent ***μ***, computed from the field-instigated charge distribution, as a power series in external electric field **F** (provided the field strength remains small), as

$$\mu_{i}\left(F\right)=\mu_{i}\left(0\right)+\underset{j}{\sum }\alpha_{ij}F_{j}+\frac{1}{2}\underset{j,k}{\sum }\beta_{\mathrm{ijk}}F_{j}F_{k}+\cdots \;.$$ (32)

In this equation, three consecutive terms on the right-hand side designate static dipole moment ***μ***_*i*_(0), dipole polarizability $\alpha_{ij}=\frac{\partial \mu_{i}}{\partial F_{j}}$ , and first-hyperpolarizability $\beta_{\mathrm{ijk}}=\frac{\partial^{2}\mu_{i}}{\partial F_{j}\partial F_{k}}$ (Mclean and Yoshimine, 1967), respectively. An alternative representation is also available in terms of field-induced energy; however, both are equivalent according to the Hellmann–Feynman theorem (Feynman, 1939).

The components of ***α***, ***β*** can be obtained using well-known finite-difference formulas (Smith, 1978):

$$\left.\begin{array}{c} \alpha_{ii}F_{i}=\frac{2}{3}\left[\mu_{i}\left(F_{i}\right)-\mu_{i}\left(-F_{i}\right)\right]-\frac{1}{2}\left[\mu_{i}\left(2F_{i}\right)-\mu_{i}\left(-2F_{i}\right)\right]\\ \alpha_{ij}F_{j}=\frac{2}{3}\left[\mu_{i}\left(F_{j}\right)-\mu_{i}\left(-F_{j}\right)\right]-\frac{1}{2}\left[\mu_{i}\left(2F_{j}\right)-\mu_{i}\left(-2F_{j}\right)\right]\\ \beta iiiF_{i}^{2}=\frac{1}{3}\left[\mu_{i}\left(2F_{i}\right)+\mu_{i}\left(-2F_{i}\right)\right]-\frac{1}{3}\left[\mu_{i}\left(F_{i}\right)+\mu_{i}\left(-F_{i}\right)\right]\\ \beta ijjF_{j}^{2}=\frac{1}{3}\left[\mu_{i}\left(2F_{j}\right)+\mu_{i}\left(-2F_{j}\right)\right]-\frac{1}{3}\left[\mu_{i}\left(F_{j}\right)+\mu_{i}\left(-F_{j}\right)\right] \end{array}\right\}.$$ (33)

Furthermore, in addition to ***α***, ***β*** tensors, for a given species, one can also compute the so-called experimentally determined quantity, the average polarizability $\left(\bar{\alpha }\right)$ , in the form of

$$\bar{\alpha }=\frac{1}{3}\left(\alpha_{zz}+\alpha_{xx}+\alpha_{yy}\right).$$ (34)

In order to get these tensors from ***μ*** of the system (expressed as a function of **F**), one needs the perturbed density matrix at various field strengths. This can be obtained from the SCF solution of Eq. 13. Hence, the core part of the Hamiltonian (symbolized by a prime) will now be altered by a relevant/appropriate field-dependent term conventionally expressed as

$$H_{\mu \nu }^{\prime core}=H_{\mu \nu }^{\text{core}}+F_{i}\langle \mu |r|\nu \rangle ,\;\;\;\;\;\;\;\;\;i\in \left\{x,y,z\right\}.$$ (35)

Here, $H_{\mu \nu }^{\text{core}}$ denotes the unperturbed core Hamiltonian mentioned above, *F*_*i*_ refers to *i*th component of **F**, and ⟨*μ*|**r**|*ν*⟩ provides the dipole moment integral related to length vector **r**. The two-body matrix elements do not change during FF calculations. Eventually, ***μ*** of a molecule can be described as follows:

$$\mu \equiv \mu_{el}=\underset{\mu \nu }{\sum }P_{\mu \nu }\langle \mu |r|\nu \rangle +\underset{a}{\sum }Z_{a}R_{a},$$ (36)

where *Z*_*a*_ and **R**_*a*_ are nuclear charge and position of atom “a”, respectively.

In the FF method, numerical accuracy is a crucial factor. The system’s dipole moment is computed in the presence of **F**, and the respective finite differences are used to approximate the derivatives. Hence, it is very sensitive with respect to **F**, and the field needs to be chosen with utmost care such that 1) it is adequately large to subdue finite-precision artifacts for a meaningful estimation of essential finite differences, particularly in the nonlinear domain for ***β*** and 2) it must also be small enough to be able to neglect the higher-order derivatives for one particular coefficient. Selection of the appropriate field strengths initiates with picking up an initial field strength (F_0_), around which other field strengths (F) are distributed. This is achieved by selecting the field distribution according to the following relation:

$$F_{n}=F_{0}\;2^{\frac{3n}{100}},$$ (37)

where *n* ranges from 0 to 160, and *F*_0_ = 0.0005 a.u. It gives a maximum field strength larger than 0.01 a.u. For a given *F*_*n*_, these properties are calculated for a fixed grid and basis set.

### 3.2 Field Sensitivity and Its Optimization

To address the delicate nature of electric field on these properties, in addition to the above procedure, we also adopted a recently proposed (Patel et al., 2017) technique, whereby the energy is fitted with respect to electric-field coefficients in the form of a rational function. This is examined by a fitting strategy for induced dipole moment in terms of the electric field as follows:

$$\mu \left(F\right)=\frac{\;a+bF+cF^{2}+dF^{3}\cdots }{\;1+BF+CF^{2}+DF^{3}\cdots }.$$ (38)

where a,b,c,d,⋯ and B,C,D,⋯ are fitting coefficients. If the denominator coefficients are set to zero, this gives rise to a generalized form of Taylor expansion. Such a recipe has the advantage that it provides a less sensitive (thus more effective) dependence on the electric field, as it enlarges the range of the field. In the FF technique, ***μ*** needs to be computed at certain field strengths. That requires a proper selection of initial field strength (*F*_0_), which is achieved here *via* a proposal put forth by Patel et al. (2017):

$$F_{n}=x^{n}F_{0},\;x=2^{\frac{p}{100}}.$$ (39)

The recommended value of *p* is 50, corresponding to a geometric progression. This was arrived on the basis of a systematic and detailed analysis of ***α*** and ***γ*** for a set of 121 and ***β*** for 91 molecules. Following Ghosal et al. (2018), an initial value of 10^−2.5^ was found to be quite appropriate. The optimal form of rational function is adopted (Patel et al., 2017) as

$$\mu \left(F\right)=\frac{\;a+bF+cF^{2}+dF^{3}}{\;1+BF+CF^{2}},$$ (40)

containing four and three terms in numerator and denominator. Now putting value of ***μ*** at **F** = 0 in the above equation leads to a trivial relation, ***μ*** (0) = *a*. The remaining unknown coefficients can be determined employing different *F*_*n*_. For five unknown coefficients, six minimum equations can be constructed (as both +*F*_*n*_ and −*F*_*n*_ used), each *F*_*n*_ giving two equations. Employing ***μ*** (0) for *a*, one may write the following matrix equation of form, **Ax** = **b**:

$$\left[\begin{array}{ccccc} -F_{0}\mu \left(F_{0}\right) & -F_{0}^{2}\mu \left(F_{0}\right) & F_{0} & F_{0}^{2} & F_{0}^{3}\\ F_{0}\mu \left(-F_{0}\right) & -F_{0}^{2}\mu \left(-F_{0}\right) & -F_{0} & F_{0}^{2} & -F_{0}^{3}\\ -xF_{0}\mu \left(xF_{0}\right) & -x^{2}F_{0}^{2}\mu \left(xF_{0}\right) & xF_{0} & x^{2}F_{0}^{2} & x^{3}F_{0}^{3}\\ xF_{0}\mu \left(-xF_{0}\right) & -x^{2}F_{0}^{2}\mu \left(-xF_{0}\right) & -xF_{0} & x^{2}F_{0}^{2} & -x^{3}F_{0}^{3}\\ -x^{2}F_{0}^{2}\mu \left(x^{2}F_{0}^{2}\right) & -x^{4}F_{0}^{4}\mu \left(x^{2}F_{0}^{2}\right) & x^{2}F_{0}^{2} & x^{4}F_{0}^{4} & x^{6}F_{0}^{6}\\ x^{2}F_{0}^{2}\mu \left(-x^{2}F_{0}^{2}\right) & -x^{4}F_{0}^{4}\mu \left(-x^{2}F_{0}^{2}\right) & -x^{2}F_{0}^{2} & x^{4}F_{0}^{4} & -x^{6}F_{0}^{6} \end{array}\right]\left[\begin{array}{c} B\\ C\\ b\\ c\\ d \end{array}\right]=\left[\begin{array}{c} \mu \left(F_{0}\right)-\mu \left(0\right)\\ \mu \left(-F_{0}\right)-\mu \left(0\right)\\ \mu \left(xF_{0}\right)-\mu \left(0\right)\\ \mu \left(-xF_{0}\right)-\mu \left(0\right)\\ \mu \left(x^{2}F_{0}^{2}\right)-\mu \left(0\right)\\ \mu \left(-x^{2}F_{0}^{2}\right)-\mu \left(0\right) \end{array}\right].$$ (41)

The solution of this equation is overdetermined, as both (+)ve and (−)ve fields are used. A least-squares method can be convenient; else, one may disregard any one equation. The current work invokes the latter, where one of the six equations is arbitrarily eliminated. The required properties can be determined from derivatives of Eq. 38 at *F* = 0:

$$\alpha =\mu^{\prime }\left(0\right)=b-aB,\;\;\;\;\;\;\;\;\;\;\;\beta =\mu^{\prime \prime }\left(0\right)=2c-2bB-2aC-2aB^{2}.$$ (42)

Further details of the method and its validation can be found in Mandal et al. (2019).

### 3.3 Numerical Tests and Convergence

This section presents some sample results to demonstrate the applicability of the above-described method. At first, a few practical points may be pointed out. All computations are executed involving norm-conserving pseudopotential at the experimental geometries taken from the NIST database (Johnson, 2016). A simple grid-optimization technique has been followed, ensuring a grid precision of at least 5 × 10^−6^ a.u., all through, at a fixed *h*_*r*_ = 0.3. It was noticed that the optimal non-uniform grid marginally differs from functional to functional. We employ the LFK basis set for this study. The properties are inspected for four representative XC functionals, namely, LDA, BLYP, PBE, and LBVWN. The SCF convergence criteria imposed in this calculation to generate the perturbed density matrix are as follows: 1) orbital energy difference between two consecutive iterations and 2) absolute deviation in a density matrix element. They both must stay below a specific predetermined threshold, which is set to 1 × 10^−8^ a.u. This assured that total energy maintains a convergence to at least this level. In order to facilitate the convergence, an unperturbed (field-free) density matrix was employed as trial input. The convergence was carefully examined with respect to all parameters, such as grid and field optimization, both in the absence and presence of the electric field.

Let us now examine the ***α*** and ***β*** tensors. Following Buckingham and Hirschfelder (1967), we have two independent components (***α***_*xx*=*yy*_, ***α***_*zz*_) associated with ***α*** and ***β*** (***β***_*xxz*=*yyz*_, ***β***_*zzz*_), for a hetero-nuclear diatomic molecule having *C*_*∞v*_ symmetry. The maximum field response toward the electron density is then found along the *z* direction as it is the molecular axis. Now, as a check, we have performed the GAMESS calculations (Schmidt et al., 1993) with default grid options, that is, 96 radial and 302 angular points for the spatial grid and 0.001 for field strength. A recent study of grid effects (based on ACG), reported in Castet and Champagne (2012), suggested a spatial grid of 99 radial and 974 angular points to be an optimal solution. It has been observed that the default option delivers results that are practically coincident with that from the finer grid; we have verified this for three diatomic molecules (HCl, HBr, and HI). Thus, for all practical purposes, the default grid suffices the current purpose.

#### 3.3.1 Comparison With Standard Packages

Now with this preamble, at first, we report the non-zero components of ***α***, ***β*** in addition to $\bar{\alpha }$ and ***μ***, of a representative test molecule, HCl in Table 1. These are supplied for all four XC functionals. We quote the reference values (except for LBVWN) acquired from GAMESS software (Schmidt et al., 1993). Comparative components for a few other selective diatomic molecules are additionally presented by Ghosal et al. (2018), which are excluded here to save space. The largest mean absolute deviation (MAD) in ***μ***_*z*_ in HCl is around 5 × 10^−5^ a.u., for PBE, whereas the LDA and BLYP results impeccably coincide with reference (in all the digits quoted) for LDA and BLYP. The ***α***, ***β*** tensors of our calculations are also similarly consistent with the reference data. For comparison, a few relevant theoretical outcomes are cited in the footnote, alongside the methods (such as higher-order perturbation theory, MCSCF, and CCSD(T)) and basis set. Also, experimental values for $\bar{\alpha }$ are additionally recorded from two different kinds of experimental strategies (Olney et al., 1997; Hohm, 2013). These values contain just the electronic part, and neither geometry relaxation in the presence of the electric field nor vibrational contribution is considered. It uncovers a fascinating fact that all three traditional functionals (LDA, BLYP, and PBE) overestimate both experimental outcomes; however, LBVWN underestimates. These conclusions are in accordance with the behavioral pattern of these functionals for $\bar{\alpha }$ in Cohen et al. (1999) and Vasiliev and Chelikowsky (2010). This verification for the diatomic hydrides goes about as a test-bed for the following arrangement of atoms and molecules. Note that because the converged properties reproduce standard GAMESS results (Schmidt et al., 1993) for all XC functionals (verified for other systems as well), these reference values are discarded hereafter.

**TABLE 1:** Static dipole moment ***μ***_*z*_ and FF $\alpha ,\bar{\alpha },\beta$ values (in a.u.) of HCl for different XC functionals. PR implies present result. More details could be found in Ghosal et al. (2018).

	XC	***μ***_*z*_		***α***_*xx*=*yy*_		***α***_*zz*_		$\bar{\alpha }$ ^a^	***β***_*xxz=yyz*_		***β***_*zzz*_	
Molecule	Functionals	PR	Referernce (Schmidt et al., 1993)	PR	Reference (Schmidt et al., 1993)	PR	Reference (Schmidt et al., 1993)	PR	PR	Reference (Schmidt et al., 1993)	PR	Reference (Schmidt et al., 1993)
HCl^b^ ^,^ ^c^ ^,^ ^d^	LDA	−0.43826	−0.43826	18.48	18.48	19.38	19.38	18.79	8.26	8.27	20.77	20.77
	BLYP	−0.42337	−0.42337	18.19	18.19	19.24	19.24	18.55	6.28	6.28	19.60	19.60
	PBE	−0.43420	−0.43425	18.05	18.04	19.01	19.01	18.37	7.19	7.19	18.91	19.89
	LBVWN	−0.45357	—	15.39	—	17.41	—	16.07	3.77	—	15.20	—

a Experimental $\bar{\alpha }$ of HCl: (i) dipole (e,e) method (Olney et al., 1997) = 16.97, (ii) refractive index method (Hohm, 2013) = 17.40, 23.78, 35.30.

b CAS (taug-cc-pVTZ) (Bishop and Norman, 1999): ***μ***_*z*_ = 0.45, ***α***_*xx*=*yy*_ = 16.86, ***α***_*zz*_ = 18.52, $\bar{\alpha }=17.41$ , ***β***_*xxz*=*yyz*_ = −0.31, ***β***_*zzz*_ = −11.32.

c CAS (qaug-sadlej) (Fernández et al., 1998): ***α***_*xx*=*yy*_ = 16.6952, ***α***_*zz*_ = 18.3361, $\bar{\alpha }=17.2422$ , ***β***_*xxz*=*yyz*_ = 0.64, ***β***_*zzz*_ = 12.71.

d CCSD(T) (KT1 basis) (Maroulis, 1998): ***μ***_*z*_ = 0.4238, ***α***_*xx*=*yy*_ = 16.85, ***α***_*zz*_ = 18.48, $\bar{\alpha }=17.39$ , ***β***_*xxz*=*yyz*_ = −0.2, ***β***_*zzz*_ = −10.7.

#### 3.3.2 Results on $\mu ,\bar{\alpha },\beta$

Now, in Table 2, we present ***μ*** for some linear and non-linear molecules covering both close and open shells of systems extending from diatomic to the hexa-atomic ones at equilibrium geometry. These correspond to the electronic part only; geometry relaxation or vibration contribution has not been incorporated. The total energies are accurately reproduced by the present calculation and not reported here. These can be found in Mandal et al. (2019). The computed zero values of components of ***μ*** in the case of non-polar molecules have been correctly produced and henceforth not discussed. The polar molecules show good overall concurrence with experimental results. For a bunch of 29 molecules, the MAD from respective experimental outcomes is 13% (for PBE, LDA) and 10% (for LBVWN, BLYP), separately.

**TABLE 2:** Permanent dipole moment of molecules for four XC functionals. All results in a.u. and taken from Mandal et al. (2019).

Molecule	***μ***				
	LDA	BLYP	PBE	LBVWN	Expt.^a^
HF	0.70315	0.68988	0.69307	0.75623	0.71604
HCl	0.43825	0.42337	0.43420	0.45357	0.42490
H_2_O	0.71610	0.69956	0.70607	0.76583	0.7278
NH_3_	0.57940	0.57091	0.57498	0.59063	0.57834
SiH_3_Cl	0.50313	0.50656	0.49827	0.58014	0.51539
CH_3_Cl	0.73076	0.73269	0.72914	0.71637	0.73571
CH_3_Br	0.71377	0.72486	0.71875	0.63353	0.71210
C_3_H_8_	0.04065	0.03925	0.03844	0.03102	0.03304

a For HCl and CHCl_3_, the dielectric method (Nelson et al., 1967); for all others, the microwave spectroscopy method (Nelson et al., 1967).

Next, we advance toward $\bar{\alpha }$ of a cross-section of atoms and molecules, in Table 3. For the sake of comparison, accessible theoretical values from the NIST database and experimental values of zero frequency (containing electronic part only) (Johnson, 2016; Miller and Bederson, 1997) are quoted for comparison. It reveals that each of the three customary functionals (LDA, BLYP, and PBE) overestimate the experimental references. However, LBVWN offers underestimation in all cases (except for Be) and thus differentiates from the other three mentioned functionals.

**TABLE 3:** Average static polarizability, $\bar{\alpha }$ for some atoms and molecules using FF KS method, for four XC functionals. All results in a.u. For details, see Mandal et al. (2019).

	$\bar{\alpha }$ (Atom)						$\bar{\alpha }$ (Molecule)				
	LDA	BLYP	PBE	LBVWN	Referernce^a^		LDA	BLYP	PBE	LBVWN	Reference^b^
Be (s1)	44.49	43.43	43.10	40.81	37.79	HF	6.24	6.25	6.15	4.88	5.09
B (P2)	22.24	21.88	21.11	18.81	20.45	HCl	18.79	18.55	18.37	16.07	17.40
O (S3)	5.62	5.48	5.47	4.21	5.41	H_2_O	10.52	10.41	10.26	8.91	9.52
Mg (S1)	76.91	75.02	74.23	70.51	71.53	NH_3_	15.43	15.31	15.08	12.59	14.61
Si (P3)	37.50	37.89	36.23	35.13	36.31	SiH_3_Cl	44.93	43.81	43.86	39.93	35.8
P (S4)	28.68	28.27	27.91	24.17	24.50	CHCl_3_	60.12	59.52	58.98	52.02	56.22
Cl (P2)	16.25	16.51	15.73	13.84	14.71	Si_2_H_6_	65.76	63.32	63.81	59.02	63.53
Xe (S1)	28.67	28.42	28.04	25.02	27.29	C_4_ $H_{6}^{c}$	59.34	59.64	58.27	53.07	54.64

a Theoretical values are from Miller and Bederson (1997), as quoted in the NIST database (Johnson, 2016).

b Zero-frequency result. For SiH_3_Cl, the dielectric permittivity method (Hohm, 2013); for all others, the refractive index method (Hohm, 2013).

Now, in this segment, we proceed to higher-order coefficients; Table 4 reports non-zero components of ***β*** (using *T* convention) for some representative molecules. It is evident that the components of a given molecule differ fundamentally from functional to functional—in some cases, including even the sign changes. One such candidate is HI, where ***β***_*xxz*_, ***β***_*yyz*_ signs for LDA, PBE functionals are opposite from those of BLYP, PBE. For a comparative understanding, a few selected high-level all-electron calculations (such as CCSD, CAS, and CCSD(T)) in elaborate basis sets (NLO-II, Sadlej, qaug-sadlej, and taug-cc-pVTZ) are provided, along with certain experiments. For clear reasons, our outcomes differ from extended calculations rather significantly. However, this is not the primary objective of this work.

**TABLE 4:** The components of ***β*** for some selected molecules for four XC functionals. All results are in a.u. See Mandal et al. (2019) for details.

Molecule	LDA	BLYP	PBE	LBVWN	LDA	BLYP	PBE	LBVWN	LDA	BLYP	PBE	LBVWN
	***β***_*xxz*_				***β***_*yyz*_				***β***_*zzz*_			
H_2_S^a^	−12.41	−14.30	−12.21	−4.93	6.96	5.80	6.07	8.78	−25.07	−27.54	−24.92	−7.31
PH_3_ ^b^	6.07	4.56	6.38	5.19	6.07	4.56	6.39	5.19	20.73	5.63	14.79	6.12
CHCl_3_ ^c^	−19.42	−18.65	−17.82	−11.75	−18.49	−17.80	−16.92	−11.42	15.31	17.06	15.94	2.17
	***β***_*xxy*_				***β***_*yzz*_				***β***_*yyy*_			
C_3_H_8_	1.04	2.99	1.69	1.82	−25.01	−25.15	−23.92	−14.13	−28.59	−26.24	−26.30	−11.06
	***β***_*xyy*_				***β***_*xzz*_				***β***_*xxx*_			
CH_3_Br	42.62	45.80	4.84	21.94	42.59	45.86	44.13	21.94	17.24	18.72	21.43	5.40
	***β***_*xxz*_ = ***β***_*yyz*_				***β***_*zzz*_							
HF^d^	−3.59	−3.24	−3.22	−1.51	−14.09	−14.04	−13.52	−9.24				
HCl^e^	8.27	6.30	7.19	3.78	20.77	19.60	18.91	15.19				
HI	−3.32	1.19	−1.80	2.39	−16.48	−12.64	−13.22	−9.22				

a CCSD (polarizability-consistent Sadlej) (Sekino and Bartlett, 1993): ***β***_*zzz*_ = 7.7, ***β***_*xxz*_ = −1.2, and ***β***_*yyz*_ = −11.7. Experimental value ⟨ ***β*** ⟩ = $\sqrt{\left(\sum_{i}\beta_{i}^{2}\right)}$ = − 10.1, ***β***_*i*_ = (1/3)*∑*_*k*_ ***β***_*ikk*_ (Sekino and Bartlett, 1993).

b CCSD (NLO-II) (Pascola et al., 2014): ⟨ ***β*** ⟩ = $\sqrt{\left(\sum_{i}\beta_{i}^{2}\right)}$ = −18.5, ***β***_*i*_ = (1/3)*∑*_*k*_ ***β***_*ikk*_.

c CCSD-QRF (d-aug-cc-pVDZ): ***β***_*HRS*_ = 15.05 and TDHF (d-aug-cc-pVDZ): ***β***_*HRS*_ = 10.02 (de Wergifosse et al., 2015); Experimental value (Hyper–Rayleigh scattering experiment) ***β***_*HRS*_ = −19.0 (Castet and Champagne, 2012); $\beta_{HRS}=\sqrt{\left(\left\langle \beta_{ZZZ}^{2}\right\rangle +\left\langle \beta_{XXZ}^{2}\right\rangle \right)}$ , corresponding to laboratory axes.

d CAS (taug-cc-pVTZ) (Bishop and Norman, 1999): ***β***_*xxz*_ = ***β***_*yyz*_ = −1.2, ***β***_*zzz*_ = −8.77. CCSD (polarizability-consistent Sadlej) (Sekino and Bartlett, 1993): ***β***_*xxz*_ = ***β***_*yyz*_ = −0.08, ***β***_*zzz*_ = −8.91. Experimental value ⟨ ***β*** ⟩ = 11.0 (Shelton and Rice, 1994).

e CAS (taug-cc-pVTZ) (Bishop and Norman, 1999): ***β***_*xxz*_ = ***β***_*yyz*_ = −0.31, ***β***_*zzz*_ = −11.32. CCSD(T) (KT1) (Maroulis, 1998): ***β***_*xxz*_ = ***β***_*yyz*_ = −0.2, ***β***_*zzz*_ = −10.7. Experimental value ⟨ ***β*** ⟩ = 9.8 (Dudley and Ward, 1985).

#### 3.3.3 Distorted Geometries

**FIGURE 1:**
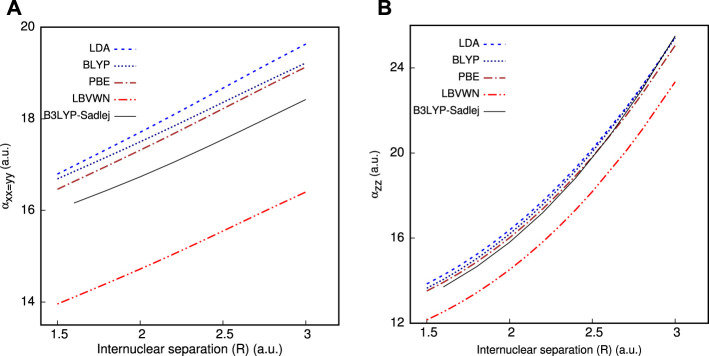
Impact of *R* on **(A)** ***α***_*xx*=*yy*_ and **(B)** ***α***_*zz*_ of HCl molecule, taken from Ghosal et al. (2018).

We now offer some illustrative results to explore the efficacy of CCG in determining non-zero components of ***μ*** and ***α***, ***β*** tensors, as functions of *R*, in Table 5. As an example, HCl is chosen with *R* ranging from 1.5 to 3.0 a.u. In general, beyond equilibrium geometry, the static correlation becomes predominant; subsequently, the role of XC functional is of utmost significance. Moreover, the role of the basis set is likewise a major factor. The computed values are in excellent concurrence with theoretical references for all XC functionals throughout the entire region. Upon closer investigation, there is an adjustment in sign in ***β***_*xxz=yyz*_ on shifting *R* from 2.5 to 3 a.u., which is quite satisfactorily captured in InDFT (Roy et al., 2019). Besides that, a comparison with all-electron calculations is additionally performed and portrayed in Figure 1, which are done using the Sadlej basis (Sadlej, 1992) and standard B3LYP functional through the GAMESS program. All functionals reproduce the qualitative shape of ***α***_*xx*=*yy*_ and ***α***_*zz*_ very well for the whole range. In both panels, PBE is the nearest to Sadlej-B3LYP results around *R*_*eq*_. While in panel (a), all plots stay well separated, a distinct crossover is recorded in panel (b) as one moves farther past *R*_*eq*_. The PBE plot in (b) tends to deviate maximum from all-electron results in the case of all functionals. Consequently, InDFT (Roy et al., 2019) can produce competitive results for ***α***_*xx*=*yy*_, ***α***_*zz*_, with more elaborate full calculations, both around and away from equilibrium. More point-by-point results and thorough analysis could be found in Mandal et al. (2019).

**TABLE 5:** Static dipole moment ***μ***_*z*_, along with FF $\alpha ,\bar{\alpha },\beta$ (in a.u.) of HCl molecule at various distorted geometries. All quantities are in a.u. More details are available in Ghosal et al. (2018).

*R*	XC	***μ***_*z*_		***α***_*xx*=*yy*_		***α***_*zz*_		***β***_*xxz*=*yyz*_		***β***_*zzz*_	
	Functional	PR	Reference (Schmidt et al., 1993)	PR	Reference (Schmidt et al., 1993)	PR	Reference (Schmidt et al., 1993)	PR	Reference (Schmidt et al., 1993)	PR	Reference (Schmidt et al., 1993)
1.5	LDA	−0.31111	−0.31111	16.80	16.80	13.85	13.85	20.29	20.29	18.59	18.59
	BLYP	−0.31210	−0.31213	16.69	16.69	13.64	13.64	19.20	19.19	16.55	16.52
	PBE	−0.32008	−0.32013	16.46	16.46	13.52	13.52	19.37	19.35	17.05	16.89
2.5	LDA	−0.45428	−0.45429	18.67	18.67	20.19	20.19	6.48	6.48	20.98	20.99
	BLYP	−0.43630	−0.43630	18.36	18.35	20.06	20.06	4.39	4.40	19.84	19.83
	PBE	−0.44800	−0.44804	18.22	18.21	19.81	19.81	5.39	5.39	19.07	19.07
3.0	LDA	−0.55506	−0.55506	19.63	19.63	25.44	25.44	−3.83	−3.82	26.01	26.01
	BLYP	−0.51364	−0.51361	19.22	19.21	25.42	25.42	−6.49	−6.47	24.69	24.68
	PBE	−0.53309	−0.53312	19.13	19.13	25.05	25.05	−4.98	−4.99	23.25	23.26

## 4 HF Exchange Through FCT

While DFT has witnessed an overwhelming number of successful applications, in general, the DFA arouses certain discomfitures. These are 1) piece-wise linearity (PWL) of total energy in fractional particle numbers (Perdew and Wang, 1992; Yang et al., 2000), 2) non-cancellation of fictitious Coulomb self-repulsion energy, often called self-interaction error (SIE) (Perdew and Zunger, 1981; Bao et al., 2018), and 3) asymptotically correct XC potential behavior at LR (Levy et al., 1984). The above three issues are not equivalent, but, to a certain extent, they are interconnected (Kronik and Kümmel, 2020). They provide crucial guidelines in the development of advanced density functionals. A prominent route through which these problems can be addressed is *via* the introduction of EEX into the picture, which may be combined empirically (Becke, 1993) or non-empirically (Perdew et al., 1996b; Guido et al., 2013), with semi-local functionals. This can improve the asymptotic nature, and, as a consequence, the SIE may get reduced significantly. Following this, the global hybrid functionals (Becke, 1993) (a classic case being B3LYP) were subsequently proposed (Adamo and Barone, 1999; Ernzerhof and Scuseria, 1999; Zhao and Truhlar, 2008); this has tremendously enhanced the chemical applicability of DFT in a large range of chemical systems (Silva-Junior et al., 2008; Mangiatordi et al., 2012). Recently, static correlation in covalent bonds has been treated with general single-determinant model functionals up to the dissociation limit (Becke, 2013; Kong and Proynov, 2015). These emerging hyper-GGA functionals involve exact exchange energy density, *e* ^x^, as a fundamental variable, requiring a higher computational cost than the global hybrid ones. Another promising route through which the above conditions can be controlled is optimal tuning of RSH functionals (Baer et al., 2010; Kronik and Kümmel, 2018).

Within the scope of basis-set expansion of MOs, HF exchange energy and related matrix elements can be calculated analytically through four-center electron repulsion integrals (ERI), when GTOs are used. This provides the following contribution to the KS-Fock matrix:

$$F_{\mu \nu }^{\text{x}\sigma }=\underset{\lambda \eta }{\sum }P_{\lambda \eta }^{\sigma }\left(\mu \lambda |\eta \nu \right)=\underset{\lambda \eta }{\sum }P_{\lambda \eta }^{\sigma }\iint \frac{\chi_{\mu ,\sigma }\left(r\right)\chi_{\lambda ,\sigma }\left(r\right)\chi_{\nu ,\sigma }\left(r^{\prime }\right)\chi_{\eta ,\sigma }\left(r^{\prime }\right)}{\left|r-r^{\prime }\right|}drdr^{\prime }.$$ (43)

Here, ERI is represented by (*μλ*|*ην*) with *μ*, *ν*, *λ*, *η* denoting the contracted AO basis, and $P_{\lambda \eta }^{\sigma }$ represents an element of single-particle spin density matrix, *P* ^*σ*^ with spin *σ*. The corresponding HF exchange energy density can be expressed as

$$\begin{array}{ccc} e_{\sigma }^{\text{x}}\left(r\right)=-\overset{\mathrm{occ}}{\underset{i}{\sum }}\overset{\mathrm{occ}}{\underset{j}{\sum }}\int \frac{\phi_{i,\sigma }^{*}\left(r\right)\phi_{j,\sigma }^{*}\left(r\right)\phi_{i,\sigma }\left(r^{\prime }\right)\phi_{j,\sigma }\left(r^{\prime }\right)}{\left|r-r^{\prime }\right|}dr^{\prime } & & \\ =-\underset{\mu \nu }{\sum }\underset{\lambda \eta }{\sum }P_{\mu \nu }^{\sigma }P_{\lambda \eta }^{\sigma }\int \frac{\chi_{\mu ,\sigma }\left(r\right)\chi_{\lambda ,\sigma }\left(r\right)\chi_{\nu ,\sigma }\left(r^{\prime }\right)\chi_{\eta ,\sigma }\left(r^{\prime }\right)}{\left|r-r^{\prime }\right|}dr^{\prime }. & & \end{array}$$ (44)

The first and second mathematical forms are written in terms of KS-occupied MO (*ϕ*_*σ*_) and AO (*χ*_*σ*_). The real form of density matrix and basis gives us the liberty to omit the complex conjugate sign. At first glance, its computational cost appears to be higher than the regular exchange energy calculation (as it requires to be computed at each grid point with four AO indices). Of late, a few proposals have been reported showing considerable computational cost lightening through a pair-atomic RI (Proynov et al., 2010; Liu et al., 2012; Proynov et al., 2012) or a semi-numerical (SNR) (Kossmann and Neese, 2009; Bahmann and Kaupp, 2015; Liu and Kong, 2017; Laqua et al., 2018) approximation.

In what follows, we present a simple, novel strategy for calculating HF exchange density, energy, and necessary matrix elements in CCG, which are significant components for some XC functionals (especially orbital-dependent ones). This takes inspiration from Liu and Kong (2017), where evaluating an intermediate quantity, such as the two-center ESP integral, is a vital step, given as follows:

$$v_{\nu \eta }\left(r\right)=\int \frac{\chi_{\nu }\left(r^{\prime }\right)\chi_{\eta }\left(r^{\prime }\right)}{\left|r-r^{\prime }\right|}dr^{\prime }=\underset{p}{\sum }\underset{q}{\sum }\int \frac{\varphi_{\nu }^{p}\left(r^{\prime }\right)\varphi_{\eta }^{q}\left(r^{\prime }\right)}{\left|r-r^{\prime }\right|}dr^{\prime }.$$ (45)

The two expressions are based on AO basis and primitive functions, $\varphi_{\nu }^{p}$ for a particular *χ*_*ν*_ (spin indices omitted for simplicity). We offer a direct, efficient numerical (NR) strategy for the accurate, reliable calculation of ESP integral. This is founded on FCT and employs an RS technique, leading to an LR and SR interaction for CIK. A critical point is the characterization of optimal RS parameter for successful mapping of CIK in CCG from first principles and not empirically. Here, it is achieved through a grid-optimization technique (Ghosal et al., 2016; Ghosal et al., 2018) with respect to the total energy in CCG through a well-defined constraint. This is helpful in the additional development of so-called RSH functionals in combination with the generalized KS theorem (Seidl et al., 1996).

### 4.1 HF Exchange Energy, Density, and Matrix Elements

This section delineates a numerical methodology for EEX energy and potential, where the former is evaluated by integrating the respective density, $e_{\sigma }^{\text{x}}\left(r\right)$ , given as

$$E_{\sigma }^{\text{x}}=\frac{1}{2}\int e_{\sigma }^{\text{x}}\left(r\right)dr.$$ (46)

Now, Eq. 44 can be rewritten as follows:

$$e_{\sigma }^{\text{x}}\left(r\right)=-\underset{\nu }{\sum }Q_{\nu }^{\sigma }\left(r\right)M_{\nu }^{\sigma }\left(r\right).$$ (47)

One may anticipate the computation of $e_{\sigma }^{\text{x}}\left(r\right)$ in three phases. The first component, $Q_{\nu }^{\sigma }\left(r\right)$ can be written as follows:

$$Q_{\nu }^{\sigma }\left(r\right)=\underset{\mu }{\sum }\chi_{\mu ,\sigma }\left(r\right)P_{\mu \nu }^{\sigma },$$ (48)

in which a simple matrix multiplication is used to combine density matrix with the AOs. The step computationally scales as $O\left(N_{g}N_{B}^{2}\right)$ , where *N*_*B*_, *N*_*g*_ refer to total number of basis functions and grid points, respectively. The next vital step pertains to the evaluation of ESP integral (contained in $M_{\nu }^{\sigma }\left(r\right)$ ), which as per Eq. 45 also scales the same way. This integral is usually performed analytically (second expression of Eq. 45) using various types of recursion relations such as Obara–Saika (OS) (Obara and Saika, 1986; Obara and Saika, 1988), Head-Gordon–Pople (Head-Gordon and Pople, 1988), or some other combinations (Liu et al., 2016). Here, we have performed this integral through the OS scheme in terms of expensive incomplete Gamma function; this is referred to as the SNR-OS method. Evidently, each ESP integral scales as $O\left(N_{g}N_{P}^{2}\right)$ , where *N*_*P*_ denotes the average number of primitive functions. The final step requires the computation of $M_{\nu }^{\sigma }\left(r\right)$ as follows:

$$M_{\nu }^{\sigma }\left(r\right)=\underset{\eta }{\sum }Q_{\eta }^{\sigma }\left(r\right)v_{\nu \eta }^{\sigma }\left(r\right).$$ (49)

This segment also scales in the same way as ESP integral calculation, but with lesser steps than the latter; in the innermost loop, only one multiplication and addition are required. This provides a NR route to evaluate the HF exchange matrix, which can be further modified as

$$\frac{\partial E_{\sigma }^{x}}{\partial P_{\lambda \eta }^{\sigma }}=F_{\mu \nu }^{\text{x}\sigma }=-\int_{r}\chi_{\mu }\left(r\right)M_{\nu }^{\sigma }\left(r\right)dr.$$ (50)

Then, the HF exchange energy and KS-Fock matrix with its contribution, can be evaluated accurately in CCG in a purely NR way *via* the following equations:

$$\begin{array}{ccc} E_{\sigma }^{\text{x}}=\frac{1}{2}h_{x}h_{y}h_{z}\underset{g}{\sum }e_{\sigma }^{\text{x}}\left(r_{g}\right), & & \\ F_{\mu \nu }^{\text{x}\sigma }=-h_{x}h_{y}h_{z}\underset{g}{\sum }\chi_{\mu }\left(r_{g}\right)M_{\nu }^{\sigma }\left(r_{g}\right). & & \end{array}$$ (51)

Now, to evaluate ESP integral in CCG, one can rewrite Eq. 45 in the form of

$$v_{\nu \eta }\left(r\right)=\int \frac{\chi_{\nu }\left(r^{\prime }\right)\chi_{\eta }\left(r^{\prime }\right)}{\left|r-r^{\prime }\right|}dr^{\prime }=\int \frac{\chi_{\mu \nu }\left(r^{\prime }\right)}{\left|r-r^{\prime }\right|}dr^{\prime }=\chi_{\nu \eta }\left(r\right)⋆v^{c}\left(r\right).$$ (52)

For simplicity, the spin indices are omitted here. The final mathematical form involves a convolution integral, with *χ*_*νη*_ representing a straightforward multiplication of two AO basis functions, and *v* ^*c*^(**r**) denoting the usual CIK. This is further simplified by invoking FCT as follows:

$$v_{\nu \eta }\left(r\right)=F^{-1}\left\{v^{c}\left(k\right)\chi_{\nu \eta }\left(k\right)\right\}\;where\;\chi_{\nu \eta }\left(k\right)=F\left\{\chi_{\nu \eta }\left(r\right)\right\}.$$ (53)

Here, *v* ^*c*^(**k**) and *χ*_*νη*_(**k**) signify Fourier integrals of CIK and AO basis functions. The key issue is obtaining a precise mapping of the former, which involves a singularity at **r** → 0. To deal with this concern, we use a simple RS strategy based on the works of Gill and Adamson (1996) and Gill et al. (1996), expanding the CIK into LR and SR parts using a suitable RS parameter (*ζ*) as follows:

$$\begin{array}{ccc} v^{c}\left(r\right)=\frac{erf\left(\zeta r\right)}{r}+\frac{erfc\left(\zeta r\right)}{r} & & \\ v^{c}\left(r_{g}\right)=v_{\mathrm{long}}^{c}\left(r_{g}\right)+v_{\mathrm{short}}^{c}\left(r_{g}\right). & & \end{array}$$ (54)

The last issue is determining an optimum value of parameter *ζ*_OT_, from first principles. This is achieved through the following relation:

$$\zeta_{\text{opt}}\equiv \underset{\zeta }{opt}\;E_{\sigma }^{\text{x}}\equiv \underset{\zeta }{opt}\;E_{\text{tot}}=\underset{N_{x},N_{y},N_{z}}{opt}E_{\text{tot}},\;\;at\;a\;\mathrm{fi}xed\;h_{r},$$ (55)

which is reminiscent of the grid-optimization strategy employed in Section 2.

It is worth noting that, every ESP integral is computed using only a collection of FFTs (two forward and one backward transformation) resulting in a $O\left(N_{g}\log N_{g}\right)$ scaling. This contrasts with quadratic scaling with respect to *N*_*P*_ (apart from *N*_*g*_), in the SNR-OS scheme. Hence, the computational cost is unaffected by the degree of contraction; but in SNR-OS, the cost grows quadratically with the degree of contraction. It is favorable for basis sets with substantial degrees of contraction, needed for a system with decent grid size.

#### 4.1.1 Computational Time of SNR-OS *Versus* NR

Now, we venture into a comparative discussion on the NR and SNR-OS schemes. Toward this pursuit, a real-time comparison of the performance in terms of the average effective CPU time for an SCF iteration of these two approaches is quoted for a representative set of molecules from Ghosal et al. (2019) in Table 6. Calculations are carried out on a system with Intel Core i7-7700 CPU (3.6 GHz) using the identical optimized grid. A study of the ratio $\left(\frac{SNR-OS}{NR}\right)$ suggests that it hovers in the range of 3.70 (Si_2_H_6_)–10.83 (CH_4_), implying that the NR route offers an advantageous scaling over SNR-OS. This is consistent with the scaling relations given previously. As demonstrated, the ease of implementation of this method is quite encouraging and can be intuited to have a fruitful application in future extensions.

**TABLE 6:** Timing (in s) comparison between NR and SNR-OS schemes for one SCF iteration for some representative systems, adopted from Ghosal et al. (2019).

Molecule	$t_{\text{CPU}}^{\text{NR}}\left(10^{2}\right)$	$t_{\text{CPU}}^{\text{SNR}-\text{OS}}\left(10^{2}\right)$	Ratio $\left(\frac{SNR-OS}{NR}\right)$	Molecule	$t_{\text{CPU}}^{\text{NR}}\left(10^{2}\right)$	$t_{\text{CPU}}^{\text{SNR}-\text{OS}}\left(10^{2}\right)$	Ratio $\left(\frac{SNR-OS}{NR}\right)$
Cl_2_	0.20	1.03	5.15	Si_2_H_6_	1.25	4.63	3.70
PH_3_	0.15	0.68	4.53	CH_3_Cl	0.33	3.00	9.09
CH_4_	0.23	2.49	10.83	SiH_3_Cl	0.76	3.00	3.95

#### 4.1.2 Orbital-Dependent Hybrid Functionals *via* RS-FCT

The strategy described above is applied in constructing three global hybrid functionals, namely, B3LYP, PBE0, and BHLYP, with a variable amount of former, as well as the traditional XC functional. The XC energy corresponding to each functional is expressed as follows:

$$E_{\text{xc}}^{\text{B}3\text{LYP}}=\left(1-a_{0}\right)E_{\text{LSDA}}^{\text{x}}+a_{0}E_{\text{HF}}^{\text{x}}+a_{\text{x}}E_{\text{B}88}^{\text{x}}+a_{\text{c}}E_{\text{LYP}}^{\text{c}}+\left(1-a_{\text{c}}\right)E_{\text{VWN}}^{\text{c}},$$ (56)

$$E_{\text{xc}}^{\text{PBE0}}=b_{0}E_{\text{HF}}^{\text{x}}+\left(1-b_{0}\right)E_{\text{PBE}}^{\text{x}}+E_{\text{PBE}}^{\text{c}},$$ (57)

$$E_{\text{xc}}^{\text{BHLYP}}=\left(1-c_{0}\right)E_{\text{LSDA}}^{\text{x}}+c_{0}E_{\text{HF}}^{\text{x}}+E_{\text{LYP}}^{\text{c}}.$$ (58)

Following (Stephens et al., 1994), *a*_0_, *a*_x_, *a*_c_ are 0.2, 0.72, 0.81 for B3LYP, whereas in case of PBE0 (Perdew et al., 1996b), *b*_0_ = 0.25. Note that, in PBE0, the contribution of HF exchange is slightly higher than B3LYP, but a higher proportion (*c*_0_ = 0.5) is assigned in BHLYP.

The pertinent LDA- and GGA-type functionals related to B3LYP, BHLYP, and PBE0 are as follows: 1) Vosko–Wilk–Nusair (VWN), the homogeneous electron gas correlation proposed in parametrization formula V (Vosko et al., 1980); 2) B88–incorporating Becke (Becke, 1988) semi-local exchange; 3) Lee–Yang–Parr (LYP) (Lee et al., 1988) semi-local correlation; and 4) Perdew–Burke–Ernzerhof (PBE) (Perdew et al., 1996a) functional for semi-local XC. Other computational details and scaling properties are available in Roy (2008a), Roy (2008b), Roy (2010b), Roy (2011), Ghosal et al. (2016), Ghosal et al. (2018), Mandal et al. (2019).

### 4.2 Analysis of Hybrid Functionals

Let us begin with the total energies for a few representative atoms and molecules in Table 7. The NR and SNR-OS results are reported for three sets of computations: HF, B3LYP, and PBE0. In all cases, the same pseudopotential, basis set, and convergence (both grid and SCF) criteria of the previous section were engaged. The highest absolute difference in energy (labeled E_diff_) between NR and SNR-OS is displayed side by side for simple comparison in all instances. With the exception of O and CH_4_, where absolute deviations are far below 0.0004 and 0.001 a.u., the overall agreement between these two sets of results is excellent, showing that the two energies are practically indistinguishable for all species. Needless to say, these energies are in close agreement with those from the standard package GAMESS (Schmidt et al., 1993). For a more detailed analysis, the interested reader may consult (Ghosal et al., 2019).

**TABLE 7:** HF, B3LYP, and PBE0 energies (a.u.) of atoms and molecules. *E*_diff_ = |*E*_NR_ − *E*_SNR-OS_|. These are adopted from Ghosal et al. (2019).

Atom	− ⟨*E*⟩								
	HF			B3LYP			PBE0		
	NR	SNR-OS	E_diff_	NR	SNR-OS	E_diff_	NR	SNR-OS	E_diff_
Be	0.96019	0.96019	0.00000	0.99386	0.99386	0.00000	0.99598	0.99598	0.00000
O	15.61720	15.61681	0.00039	15.80556	15.80549	0.00007	15.80351	15.80341	0.00010
Ge	3.59814	3.59814	0.00000	3.67466	3.67466	0.00000	3.68693	3.68693	0.00000
CH_4_	7.78878	7.78888	0.00010	8.00843	8.00846	0.00003	8.02684	8.02686	0.00002
SiH_3_Cl	20.19863	20.19862	0.00001	20.58442	20.58441	0.00001	20.61885	20.61885	0.00000
Si_2_H_6_	10.93377	10.93377	0.00000	11.28249	11.28249	0.00000	11.31207	11.31207	0.00000

Now, the highest occupied molecular orbital (HOMO) energies are investigated in atoms and molecules for some selected cases in Tables 8 and 9. These are collected from Ghosal et al. (2019), where a broader set of results are available. In addition to the three functionals of the (Table 7), here we also include BHLYP and experimental results. The latter table contains some *π*-electron molecules (simple, aromatic, and conjugated), where the fundamental gap (difference in energy between HOMO and LUMO) plays an important role. Accurate knowledge of such orbital energies is required for a satisfactory estimation of such gaps. As the outcomes of the NR and SNR-OS schemes are almost identical, we only proceed with the former. A comparison with available theoretical and experimental results reveals that HF HOMO energies (which do not incorporate correlation or relaxation effects) are better than any of the four DFT functionals investigated in terms of agreement with the experiment. This is generally true for a larger data set (Ghosal et al., 2019). Furthermore, it is interesting to note that, with the increase in the fractional contribution of HF exchange (which plays a key role in determining accurate asymptotic behavior at LR) in the hybrid functionals, the deviation falls (e.g., from B3LYP to BHLYP). This could be beneficial in larger systems that require a highly contracted basis, although just a moderate size of grid suffices for the purpose. The precision and ease with which it can be implemented augurs well for its future use in the development of RSH functionals, which may eventually lead to a comprehensive view of HF exchange in the asymptotic limit, bridging theoretical and experimental results.

**TABLE 8:** Negative HOMO energies, − *ϵ*_HOMO_ (in a.u.) for atoms and molecules using HF, B3LYP, PBE0 XC functionals. For details, consult Ghosal et al. (2019).

Atom	− *ϵ*_HOMO_(a.u.)					Molecule	− *ϵ*_HOMO_(a.u.)				
	HF	B3LYP	PBE0	BHLYP	Expt.^a^		HF	B3LYP	PBE0	BHLYP	Expt.^b^
Be	0.3090	0.2291	0.2387	0.2650	0.3426	Cl_2_	0.4786	0.3274	0.3433	0.3947	0.4219
S	0.3631	0.2506	0.2625	0.3043	0.3807	PH_3_	0.3849	0.2675	0.2781	0.3200	0.3626
Ga	0.2058	0.1263	0.1381	0.1590	0.2205	CH_4_	0.5416	0.3882	0.4013	0.4555	0.4998
Ge	0.2844	0.1825	0.1974	0.2232	0.2903	SiH_3_Cl	0.4509	0.3149	0.3288	0.3754	0.4281
As	0.3665	0.2426	0.2605	0.2910	0.3607	Si_2_H_6_	0.4068	0.3043	0.3152	0.3516	0.3870
Se	0.3319	0.2337	0.2451	0.2815	0.3584	P_4_	0.3844	0.2921	0.3075	0.3362	0.3381

a Optical spectroscopy (Johnson, 2016).

b Photo-electron spectroscopy (Johnson, 2016).

**TABLE 9:** Negative HOMO energies, − *ϵ*_HOMO_ (in a.u.) for selected *π*-electron molecules using HF, B3LYP, PBE0, and BHLYP XC functionals. Further details are available in Ghosal et al. (2019).

Molecule	− *ϵ*_HOMO_(a.u.)					
	HF	B3LYP	PBE0	BHLYP	Theory (Johnson, 2016)^a^	Expt.^b^
Ethylene	0.3686	0.2649	0.2796	0.3140	0.376^b^	0.3859
Propene	0.3544	0.2503	0.2645	0.2994	0.354^b^	0.3565
1,3-Butadiene (E)	0.3188	0.2308	0.2444	0.2734	0.332^b^	0.3333

a CCSD result using cc-PVTZ basis.

b Photo-electron spectroscopy (Johnson, 2016).

## 5 OT-RSH Functionals

This section presents an outgrowth of the prior work described earlier. The OT-RSH functionals perform remarkably well in resolving some of the important issues in connection with DFAs (detailed in Section 6). Generally, this is based on a partitioning of CIK into SR and LR parts, using an RS operator, *g*(*γ*, **r**), and an RS parameter, *γ*, as follows:

$$\frac{1}{r}=\frac{\tilde{g}\left(\gamma ,r\right)}{r}+\frac{g\left(\gamma ,r\right)}{r},$$ (59)

where $\tilde{g}\left(\gamma ,r\right)$ denotes the complementary RS operator. Historically, this was introduced for the first time (Leininger et al., 1997) in context of multi-resolution CI, where dynamical correlation hardly impacts LR interactions due to fast decaying features. In this scenario, *γ* plays a central role in adjusting the EEX contribution from SR to LR region for a certain *g*(*γ*, **r**). For a system, usually these two regions are treated separately: the SR region is represented using a revised inter-electronic distance-dependent local/semi-local DFA, while the LR sector, by EEX with *g*(*γ*, **r**)/**r** rectification. Based on the partitioning scheme, the XC energy can be obtained as follows:

$$E_{\text{xc}}=a_{\text{eex}}^{\text{sr}}E_{\text{eex}}^{\text{sr}}\left(\gamma \right)+\left(1-a_{\text{eex}}^{\text{sr}}\right)E_{\text{dfa}}^{\text{x, sr}}\left(\gamma \right)+b_{\text{eex}}^{\text{lr}}E_{\text{eex}}^{\text{lr}}\left(\gamma \right)+\left(1-b_{\text{eex}}^{\text{lr}}\right)E_{\text{dfa}}^{\text{x, lr}}\left(\gamma \right)+E_{\text{dfa}}^{\text{c}},$$ (60)

where $E_{\text{eex}}^{\text{sr}}$ , $E_{\text{eex}}^{\text{lr}}$ signify EEX energy contribution, whereas $E_{\text{dfa}}^{\text{x, sr}}$ , $E_{\text{dfa}}^{\text{x, lr}}$ represent DFA exchange, at SR and LR segments. A certain set of ( $a_{\text{eex}}^{\text{sr}}$ , $b_{\text{eex}}^{\text{lr}}$ ) characterizes a particular mode of partitioning for a given *g*(*γ*, **r**). RSH functionals with $b_{\text{eex}}^{\text{lr}}=1$ offer an asymptotically correct XC potential at LR region. Furthermore, choosing an optimal $a_{\text{eex}}^{\text{sr}}$ strikes a fine balance between EEX and dynamical correlation. As a result, these functionals are not fully free from SIE and also, if not tuned optimally for a desired system, do not follow the PWL condition. This occurs mainly due to a pre-defined default *γ*, generally obtained semi-empirically by fitting some reference data (Iikura et al., 2001; Tawada et al., 2004; Yanai et al., 2004; Lange et al., 2008).

In OT parlance, *γ* is usually determined from first principles by imposing Koopmans’ theorem (Salzner and Baer, 2009). It helps satisfy PWL conditions, makes XC potential asymptotically correct at the LR region, and preserves the size-dependency of *γ* on *ρ*. Consequently, these functionals improve properties that are rooted in orbital energies, such as vertical ionization energy (IE), fundamental gap, electron affinity (EA), charge-transfer (CT) excitation, optical gap, and Rydberg excitation (Livshits and Baer, 2007; Stein et al., 2010). However, these are hard to maintain with a universal *γ* (Baer et al., 2010). In recent years, techniques based on electron localization function and localized orbital locator have been attempted, which necessitates only one single SCF calculation (Borpuzari and Kar, 2017; Wang and Zhang, 2018). Also, a self-consistent OT-RSH approach (Tamblyn et al., 2014), based on a minimization of inter-atomic forces, has been reported as well; it produces better geometries and vibrational modes.

Following the general framework of RSH functionals presented in Eq. 60, three distinct types are considered in this rubric which obey a certain well-established mode of partitioning. The first one is the long-range correction (LC) scheme (Iikura et al., 2001) which looks like this

$$\left.\begin{array}{c} E_{\text{xc}}^{\text{LC}}=E_{\text{dfa}}^{\text{x, sr}}\left(\gamma \right)+E_{\text{eex}}^{\text{lr}}\left(\gamma \right)+E_{\text{dfa}}^{\text{c}},\\ g\left(\gamma ,r\right)=erf\left(\gamma r\right)\;and\;\tilde{g}\left(\gamma ,r\right)=erfc\left(\gamma ,r\right). \end{array}\right\}$$ (61)

The second one is termed as Coulomb-attenuating method (CAM) approach (Yanai et al., 2004), originally introduced utilizing a more general form of *g*(*γ*, **r**) as follows:

$$\left.\begin{array}{c} g_{\alpha ,\beta }\left(\gamma ,r\right)=\alpha +\beta \;erf\left(\gamma r\right)\;and\;{\tilde{g}}_{\alpha ,\beta }\left(\gamma ,r\right)=1-\left[\alpha +\beta \;erf\left(\gamma ,r\right)\right],\\ 0\leq \alpha +\beta \leq 1,\;0\leq \alpha \leq 1,\;and\;0\leq \beta \leq 1. \end{array}\right\}$$ (62)

The *α* parameter ensures the EEX contribution over the whole range by a factor of *α*, whereas the *β* parameter is responsible for the incorporation of DFA throughout the complete range by a factor of 1 − (*α* + *β*). In the particular scenario of *α* = 0, *β* = 1, the CAM approach gives rise to the previously mentioned LC scheme. These two parameters are related to $a_{\text{eex}}^{\text{sr}}$ in a rather difficult way. The third one, considered here, is called the long-range-corrected (LRC) (Rohrdanz et al., 2009) one, including an additional parameter in $E_{\text{eex}}^{\text{sr}}$ as

$$\left.\begin{array}{c} E_{\text{xc}}^{\text{LRC}}=a_{\text{eex}}^{\text{sr}}E_{\text{eex}}^{\text{sr}}\left(\gamma \right)+\left(1-a_{\text{eex}}^{\text{sr}}\right)E_{\text{dfa}}^{\text{x, sr}}\left(\gamma \right)+E_{\text{eex}}^{\text{lr}}\left(\gamma \right)+E_{\text{dfa}}^{\text{c}},\\ g\left(\gamma ,r\right)=erf\left(\gamma r\right)\;and\;\tilde{g}\left(\gamma ,r\right)=erfc\left(\gamma ,r\right). \end{array}\right\}$$ (63)

This extra parameter $a_{\text{eex}}^{\text{sr}}$ accounts for the incorporation of a certain particular amount of $E_{\text{eex}}^{\text{sr}}$ by a factor $a_{\text{eex}}^{\text{sr}}$ . In a special case, when $a_{\text{eex}}^{\text{sr}}=0$ , LRC leads to LC. Several other partitioning schemes and different *g*(*γ*, **r**) have been reported in the literature, primarily to deal with accurate thermochemistry and reaction height (Chai and Head-Gordon, 2008a; Chai and Head-Gordon, 2008b; Peverati and Truhlar, 2012b; Vikramaditya et al., 2018; Chan et al., 2019).

To properly incorporate $E_{\text{dfa}}^{\text{x, sr}}$ in RSH functionals, a number of schemes were proposed, such as adiabatic connection theorem (Baer and Neuhauser, 2005; Cohen et al., 2007), model exchange hole (Iikura et al., 2001; Henderson et al., 2008), and exchange energy density (Chai and Head-Gordon, 2008b; Lin et al., 2012). The present work invokes the framework of Iikura et al. (2001), involving a modified Fermi wave vector in exchange enhancement factor, applicable to any LDA or GGA type DFAs. Later, this was also utilized for CAM-B3LYP (Yanai et al., 2004) through a general *g*(*γ*, **r**), defined in Eq. 62. In this way, the SR GGA-exchange energy can be put forth as

$$\left.\begin{array}{c} E_{\text{gga}}^{\text{x, sr}}=-\frac{1}{2}\underset{\sigma }{\sum }\int \rho_{\sigma }^{\frac{4}{3}}K_{\text{gga},\sigma }^{\text{x, sr}}\;dr,\\ K_{\text{gga},\sigma }^{\text{x, sr}}=K_{\text{gga},\sigma }^{\text{x}}\left[\left(1-\alpha \right)-\beta \left\{\frac{8}{3}a_{\sigma }\left[\sqrt{\pi }\;erf\left(\frac{1}{2a_{\sigma }}+2a_{\sigma }\left(b_{\sigma }-c_{\sigma }\right)\right)\right]\right\}\right],\\ a_{\sigma }=\frac{\gamma }{2K_{\text{gga},\sigma }^{\text{f}}},b_{\sigma }=\exp\left(-\frac{1}{4a_{\sigma }^{2}}\right),c_{\sigma }=2a_{\sigma }^{2}b_{\sigma }+\frac{1}{2},K_{\text{gga},\sigma }^{\text{f}}={\left(\frac{9\pi }{K_{\text{gga},\sigma }^{\text{x}}}\right)}^{\frac{1}{2}}\rho_{\sigma }^{\frac{1}{3}}, \end{array}\right\}$$ (64)

where $K_{\text{gga},\sigma }^{\text{x}}$ signifies the enhancement factor. The average relative momentum for GGA, $K_{\text{gga},\sigma }^{\text{f}}$ , is used to define the modified GGA-enhancement factor, $K_{\text{gga},\sigma }^{\text{x, sr}}$ . One can see that Eq. 64 reproduces the original GGA DFA, when *γ* = *α* = 0. The respective potential is evaluated using $K_{\text{gga},\sigma }^{\text{x, sr}}$ , as it was employed for standard GGA DFAs (Johnson et al., 1993). More detailed discussion on SR DFAs can be found in the literature (Iikura et al., 2001; Baer and Neuhauser, 2005; Cohen et al., 2007; Chai and Head-Gordon, 2008b; Henderson et al., 2008; Lin et al., 2012).

Now, we proceed for the discussion on OT-RSH functionals and properties derived from them. Three different kinds of RSH functionals (LC, CAM, and LRC) are employed in our calculations. As in the original articles, the segmentation mode and RS operator remain unaltered. Here, however, *γ*_OT_ is determined following the strategy expressed as

$$\gamma_{\text{OT}}\equiv \;\underset{N_{x},N_{y},N_{z}}{opt}E_{\text{tot},\gamma },\;at\;\mathrm{fi}xed\;h_{r}.$$ (65)

This optimization technique does not require any fitting scheme. Through the characteristic length of a system, this procedure satisfies the size-dependency principle. In InDFT (Roy et al., 2019), these are implemented for five representative sets of functionals having a varying amount of SR/LR EEX with SR DFA exchange and traditional correlation functional. We consider the LC-BLYP (Tawada et al., 2004) and LC-PBE (Perdew et al., 1996a; Iikura et al., 2001) functionals from the LC-hybrid group with *γ* = 0.33 and *γ* = 0.30, respectively. Furthermore, for the CAM-hybrid group, CAM-B3LYP (Yanai et al., 2004) with *α* = 0.19, *β* = 0.46, *γ* = 0.33 and CAM-PBE0 (Lange et al., 2008) with *α* = 0.25, *β* = 0.75, *γ* = 0.30 are utilized. With slight modifications, the original LRC-*ω*PBEh functional (Rohrdanz et al., 2009) with *a* ^x, sr^ = 0.2, *γ* = 0.2 is employed for the LRC-hybrid group. Here, it is designated as LRC-*ω*PBEh^⋆^. To distinguish it from the original, it is superscripted with ⋆. The sole difference is about the construction of the SR DFA exchange. All the parameters are left unchanged as in the original paper, except for *γ*, which is represented by the subscript “ot.” B3LYP and PBE0, the two global hybrid functionals with a configurable quantity of EEX energy and a conventional DFA, are also compared side by side (Stephens et al., 1994; Perdew et al., 1996b). For ease of discussion, the five functionals (LC-BLYP, CAM-B3LYP, LC-PBE0, CAM-PBE0, and LRC-*ω*PBEh^⋆^) are categorized into two distinct blocks: B3LYP (containing B88 exchange and LYP correlation) and PBE0 (PBE exchange and correlation) types.

### 5.1 Ionization Energy

It is well-known that even if we have the EEX potential, the physical interpretation of KS frontier orbitals is not straightforward; the only exception is the HOMO. The ionization energy of a system can be assigned utilizing a KS analog to Koopmans’ theorem in HF theory and can be written in the form of

$$IE=-\epsilon_{\text{HOMO}}.$$ (66)

When it comes to LDA or GGA-type DFAs, Eq. 66 underestimates HOMO energy. Moreover, this will not work for other functionals outside the KS regime, especially those that interest us. The RSH functionals have, in principle, correct asymptotic behavior in the LR region, but the (G)KS version of Koopmans’ theorem is required to fully capture the essence of HOMO and its energy. It is proved that, for a selected case of an EEX operator, it is still possible to spot the (G)KS HOMO energy with − IE(*M*) (Görling and Levy, 1997), and, accordingly,

$$IE=-\epsilon_{\text{HOMO}}^{\gamma }.$$ (67)

Like KS mapping, the (G)KS map is not unique. When the RSH functional is considered, any choice of *γ* can generate a viable approximation of the (G)KS map. The apparent question is whether the (G)KS HOMO energy with − IE(*M*) can be accurately approximated by the RSH functional with a fixed value of *γ*, regardless of the system we are interested in. Hence, the comparison of (G)KS HOMO energy with experimental − IE(*M*) is a good check in determining *γ*_OT_ *via* Eq. 55. For that, the calculated negative (G)KS HOMO energies of a few atoms and molecules for 12 functionals are presented in Table 10. More detailed results are offered in Ghosal and Roy (2022a). It suggests that, for all the functionals, these are close to each other, but a comparison with experimental values indicates an underestimation of obtained results. The fruitfulness of OT-RSH functionals can be probed through a quantity designed as, ϒ = MAE(RSH)/MAE(OT-RSH); this ratio ϒ suggests the reduction in error relative to its unoptimized counterpart (fixed *γ*). Here, MAE signifies the mean average error. Based on this measure, the five OT functionals from both blocks, for atoms, can be arranged in the following descending order: ${ϒ}_{LRC_{\omega }PBEh_{ot}},{ϒ}_{CAM-PBE0_{ot}},{ϒ}_{LC-PBE_{ot}},{ϒ}_{LC-BLYP_{ot}},{ϒ}_{CAM-B3LYP_{ot}}$ . However, for molecules, the arrangement is bit different and the descending order of performance is ${ϒ}_{LRC_{\omega }PBEh_{ot}},{ϒ}_{LC-PBE_{ot}},{ϒ}_{CAM-PBE0_{ot}},{ϒ}_{LC-BLYP_{ot}},{ϒ}_{CAM-B3LYP_{ot}}$ . For easy understanding, a subscript “ot” has been added to identify the respective OT functionals. It is seen that, generally, the PBE0 block performs better than B3LYP. Also, within a given block, LC functionals perform better than CAM. Perhaps this is because the auxiliary parameters (*α*, *β*, *a* ^*x*,*sr*^) may have some sensitivity during the self-consistent tuning process, which are kept unaltered. Note that, during optimization of *γ*_OT_, its compatibility with other auxiliary parameters should be taken care of. This is not examined yet and remains a matter for future investigation. In any case, however, the accuracy of RSH functionals is always improved by OT-RSH functionals irrespective of the system or block under consideration.

**TABLE 10:** Ionization energies, − *ϵ*_HOMO_ for selected atoms and molecules in eV, adopted from Ghosal and Roy (2022a).

System	B3LYP	LC-BLYP	LC-BLYP_ot_	CAM-B3LYP	CAM-B3LYP_ot_	PBE0	LC-PBE	LC-PBE_ot_	CAM-PBE0	CAM-PBE0_ot_	LRC-*ω*PBEh^⋆^	LRC-*ω*PBEh^⋆^_ot_	Expt.^a^
Atom													
Be	6.23	8.52	8.50	7.64	7.63	6.50	8.58	8.67	8.71	8.78	8.23	8.75	9.32
O	8.83	11.42	12.97	10.77	11.49	9.19	11.13	13.00	12.10	13.50	10.95	13.40	13.62
Si	5.27	7.67	7.72	6.78	6.80	5.68	7.81	8.01	8.06	8.22	7.46	8.18	8.15
S	6.82	9.37	9.82	8.49	8.69	7.14	9.34	10.00	9.75	10.24	8.97	10.19	10.36
Ge	4.97	7.27	7.32	6.41	6.43	5.37	7.44	7.62	7.66	7.79	7.12	7.76	7.90
Br	8.19	10.78	11.10	9.87	10.02	8.56	10.77	11.30	11.20	11.60	10.41	11.54	11.81
Molecule													
N_2_	11.48	14.25	14.81	13.45	13.71	11.83	13.94	14.82	14.88	15.54	13.71	15.40	15.60
NaCl	5.79	8.37	7.67	7.44	7.12	6.12	8.33	7.81	8.79	8.45	8.26	8.33	9.80
H_2_S	7.12	9.77	10.11	8.84	8.99	7.48	9.74	10.29	10.18	10.60	9.37	10.54	10.48
CH_4_	10.48	13.19	13.78	12.30	12.57	10.85	13.06	13.91	13.68	14.32	12.73	14.24	13.6
CH_3_Cl	8.02	10.67	11.04	9.76	9.94	8.39	10.60	11.20	11.14	11.59	10.27	11.51	11.29
C_2_H_4_	7.27	10.00	10.18	8.95	9.03	7.67	10.06	10.41	10.34	10.60	9.62	10.56	10.51
Si_2_H_6_	8.23	10.62	10.59	9.76	9.74	8.54	10.62	10.75	10.94	11.04	10.30	10.98	10.53

a Optical spectroscopy for the atom (Johnson, 2016). Photo-electron spectroscopy for the molecule (Johnson, 2016).

### 5.2 Fundamental Gap

We now proceed to report some properties, which are challenging within DFT, mostly due to the inaccurate description of employed functionals. With that in mind, the estimated (G)KS gap along with the experiment (Johnson, 2016) is tabulated in Table 11 for all functionals for a few illustrative atoms. These are collected from a detailed report available in Ghosal and Roy (2022a). It indicates that, for all functionals, these are comparable to each other except for LC-PBE_ot_. Taking the same measure as earlier, the five functionals in descending order of performance are as follows: LRC-*ω*PBEh^⋆^_ot_, LC-PBE_ot_, LC-BLYP_ot_, CAM-PBE0_ot_, and CAM-B3LYP_ot_. On the contrary, if we compare the MAE, then OT-RSH (LC) functionals seem to do better than OT-RSH (LRC) and OT-RSH (CAM). As found in the previous case, here also, CAM-PBE0 tends to be more accurate than CAM-B3LYP. Thus, these conclusions are in accordance with the earlier findings. For all species, the overall performance of OT-RSH functionals is better than that of RSH. Amongst them, LRC-*ω*PBEh^⋆^_ot_ and LC-BLYP_ot_ exhibit excellent performance. Note that the above calculations are done with a pseudo-valence basis and SR LDA/GGA exchange. As a result, there is a substantial prospect of additional improvement, employing an all-electron basis set, including modern SR exchange functionals.

**TABLE 11:** (G)KS gap *vs*. experimental fundamental gap for selected atoms in eV. Results are adopted from Ghosal and Roy (2022a).

Atom	B3LYP	LC-BLYP	LC-BLYP_ot_	CAM-B3LYP	CAM-B3LYP_ot_	PBE0	LC-PBE	LC-PBE_ot_	CAM-PBE0	CAM-PBE0_ot_	LRC-*ω*PBEh^⋆^	LRC-*ω*PBEh^⋆^_ot_	Exp. (Johnson, 2016)
B	2.57	7.38	7.88	5.54	5.78	2.84	6.95	7.78	7.58	8.20	6.31	8.12	8.02
O	4.40	9.32	12.13	7.98	9.28	5.33	8.93	12.37	10.91	13.44	8.81	13.23	12.18
Si	1.75	6.49	6.59	4.59	4.63	2.08	6.20	6.55	6.62	6.88	5.55	6.82	6.76
Cl	2.46	7.78	9.13	5.87	6.49	3.10	7.42	9.22	8.48	9.82	6.78	9.70	9.36
Se	1.92	6.90	7.39	4.96	5.18	2.50	6.78	7.59	7.35	7.96	6.08	7.89	7.73
Br	2.04	7.24	7.83	5.26	5.54	2.61	6.96	7.93	7.72	8.45	6.26	8.35	8.32

### 5.3 Fractional Charge

Another important measure of success of DFT is a proper description of fractional charge systems. According to the PWL condition (Perdew et al., 1982), for the ground-state energy of systems (of M electrons) with fractional number of electrons (*δ*), the energy *versus* *δ* curve should be a straight line connecting the values at integer. It can be expressed as

$$\left.\begin{array}{c} E_{\text{frac}}\left(N\right)=E\left(N\right)-E_{\text{PWL}}\left(N\right),\;N=M+\delta ,\\ E_{\text{PWL}}\left(N\right)=\left(1-\delta \right)E\left(M\right)+\delta E\left(M+1\right),\;0\leq \delta \leq 1,\\ E_{\text{PWL}}\left(N\right)=\left(1+\delta \right)E\left(M\right)-\delta E\left(M-1\right),\;-1\leq \delta \leq 0, \end{array}\right\}$$ (68)

where *E*(*N*) and *E*_PWL_(*N*) define the energy and PWL interpolation of energy for fractional number of electrons, respectively. Here, *E*_frac_ is a measure of deviation from PWL behavior. Depending on the choice of range, two different cases arise; when *E*_frac_ < 0, the curve is convex and it is concave for *E*_frac_ > 0. The well-known DFAs meet certain challenges, resulting in a smooth convex curve, whereas EEX demonstrates reverse trend. RSH functionals which comprise these two ingredients have shown improvement in this direction (Mori-Sánchez et al., 2006).

According to Janak’s theorem (Janak, 1978), for (G)KS HOMO, the change in *ϵ*_HOMO_ as a function of fractional occupation number should be straight line. This is then followed by a finite jump at integer point (due to derivative discontinuity). After that again, the variation should be a straight line till the following integer point. With this realization, fractional occupation number *n*_*i*_, is introduced in density (ignoring degeneracy in HOMO and spin indices) as

$$\rho \left(r\right)=\underset{i}{\sum }n_{i}|\phi_{i}\left(r\right){|}^{2},n_{i}\left\{\begin{array}{cc} 0,\; & i>i_{\max}\\ \delta ,\; & i=i_{\max}\\ 1,\; & i<i_{\max} \end{array}\right.,$$ (69)

where *i* *_max_* corresponds to the HOMO level.

**FIGURE 2:**
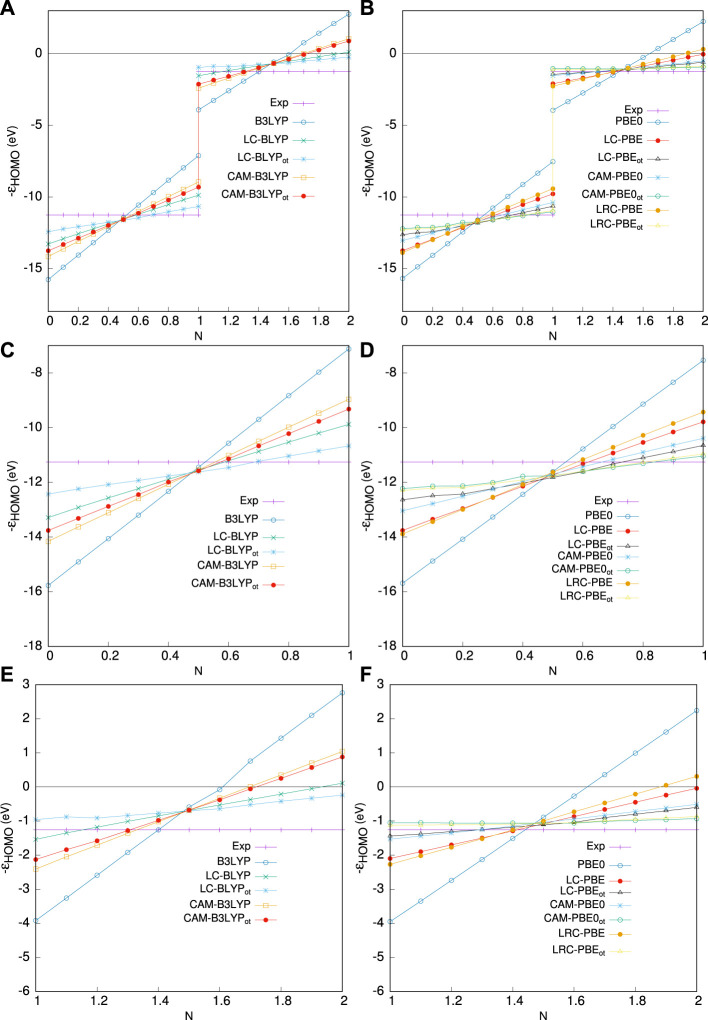
Performance of various functionals on fractional occupation in C atom. The left panel shows HOMO energy as a fraction of occupied *p*-electron number for **(A)** 0, ≤ *N* ≤ 2, **(C)** 0, ≤ *N* ≤ 1, **(E)** 1 ≤ *N* ≤ 2, for B3LYP block functionals. Right panels **(B,D,F)** refer to the PBE0 block, taken from Ghosal and Roy (2022a).

In Figure 2, we illustrate the relative performance of OT-RSH for the C atom as a test case. The pattern in the ranges −1 ≤ *δ* ≤ 0(0 ≤ *N* ≤ 1) and 0 ≤ *δ* ≤ 1(1 ≤ *N* ≤ 2) is obtained from experimental IE and EA, respectively. Three respective regions in the upper panel are 0, ≤ *N* ≤ 2 (a), 0, ≤ *N* ≤ 1 (c), and 1 ≤ *N* ≤ 2 (e), containing functionals from the B3LYP block, whereas the lower block corresponds to PBE0 functionals, in panels (b), (d), and (f). Once again, in all cases, OT-RSH shows its superiority over the respective RSH functionals. A deeper analysis of panels (c) and (e) reveals that LC-BLYP_ot_ is quite similar to the straight-line behavior in both regions, and in 1 ≤ *N* ≤ 2, it performs significantly better. From panels (d) and (f), it follows that LRC-*ω*PBEh^⋆^_ot_ and CAM-PBE0_ot_ behave similarly. In region 1 ≤ *N* ≤ 2, their performance is very close to the experiment with a tiny overall positive shift in energy. When two blocks are compared, OT-RSHs (PBE0) seem to be substantially better than OT-RSH (B3LYP); more precisely, the CAM-PBE0_ot_ outperforms the CAM-B3LYP_ot_. Based on all these facts, the five OT functionals can now be organized in declining order of performance as follows: LRC-*ω*PBEh^⋆^_ot_ ≈ CAM-PBE0_ot_ > LC-PBE_ot_ ≈ LC-BLYP_ot_ > CAM-B3LYP_ot_.

## 6 Excitation Energies

DFT has feathers in its cap as regards its application to a vast array of electronic systems in ground states. However, it suffers in the case of excited states due to several non-trivial reasons. In today’s time, the most popular approach with reasonable negotiation between accuracy and efficiency is the so-called time-dependent (TD) DFT within the linear response framework, which is a TD variant of KS-DFT. While it is, in principle, an exact method, its success mainly lies in the DFA employed and, specifically, the magnitude to which XC energies are impacted. Despite its numerous successful applications, it faces difficulties in characterizing phenomena such as double excitation, charge transfer, and Rydberg excitation. Here, we offer a simple route for accurate calculation of excited states within a time-independent approach; specifically, we are interested in the ΔSCF method (Ziegler et al., 1977; Kowalczyk et al., 2011). This uses a standard SCF iteration with the non-Aufbau occupation at each iteration using the ground-state functional. From a computational perspective, it is more convenient than linear response TDDFT due to its favorable ground-state-like scaling. Even though it provides a fair enough estimation of excitation energies, it has a tendency to variational collapse. Several sophisticated schemes, such as constrained-DFT (Ziegler et al., 2012; Barca et al., 2014; Ramos and Pavanello, 2016), methods connected to meta dynamics, and gentlest ascent dynamics (Li et al., 2015), have been quite popular in alleviating these issues.

In photo-induced electronic excitation, the lowest singlet excited energies play a crucial role. In principle, due to its multi-determinantal nature, the standard DFT cannot be used on such occasions. However, the calculation of triplet states is quite straightforward. An economical way to compute optical gaps within time-independent DFT in large molecules was introduced lately (Becke, 2016; Becke, 2018a; Becke, 2018b; Becke et al., 2018). For estimating the lowest single-particle excitation energies, the basic model employs a term known as the correlated STS energy. In essence, it involves two independent single-determinant DFT calculations: one for a closed-shell ground state and the other for the lowest triplet state with an open shell. This also requires evaluating a simple 2e^−^ integral (Coulomb self-energy) related to the HOMO-LUMO transition. Interestingly, this strategy is free from concerns involved in standard DFA applied to a triplet excited state, defined by a single Slater determinant and represented by a Fermi hole. As a result, the correlated STS energy Δ*E*_STS_ is the major element that could possibly be dealt with using 1) the adiabatic connection theorem (Becke, 2016) and 2) the virial theorem (Becke, 2018a). In a way, this is altogether a non-empirical approach that circumvents the configuration mixing.

We employ these aforementioned approaches to find the excitation energies in molecules by the above approach. The idea is to apply the FCT in conjunction with CIK to deal with the relevant 2e^−^ integral numerically. Also, to analyze its suitability and efficacy, the scheme is adopted to characterize a few properties in molecules of two different genres. First, we consider the organic chromophores, which are extremely significant in nature, showing a photo-luminescence property; a few prominent examples are photosynthesis (Murata, 1969), vision (Palczewski, 2012), and bio-luminescence (Navizet et al., 2011). These materials have wide applications in the development of unique technologies, such as organic light-emitting devices (Mitschke and Bäuerle, 2000; Forrest, 2004), fluorescent sensors (Hou et al., 2015; Li et al., 2016), organic solar cells (Taouali et al., 2018; Khan et al., 2020), medical imaging (Kundu et al., 2009; Li et al., 2016), and laser (Chénais and Forget, 2012). Another class of molecules is charge-transfer (CT) complexes, which form a distinct class of inter- and intra-molecular compounds. These molecules are characterized by the presence of a certain low-energy transition with a relatively strong oscillator strength. According to Mulliken’s quantum theory (Mulliken and Person, 1962; Mulliken and Person, 1969), the ground state of these complexes is typically a resonance hybrid wave function composed of an interacting donor (D) and acceptor (A) that can be expressed as the total of the terms, namely, neutral (DA) and dative (D^+^ A^−^, D^−^ A^+^) states as given in Eq. 70. A partial ground state charge transfer occurs when an electron is transported from donor to acceptor (D^+^ A^−^) and acceptor to donor (D^−^ A^+^). Such complexes have a wave function that looks like (Winget and Brédas, 2011)

$$\Psi \left(D,A\right)=a\Psi \left(DA\right)+\underset{i}{\sum }b_{i}\Psi \left(D^{+},A^{-}\right)+\underset{i}{\sum }c_{i}\Psi \left(D^{-},A^{+}\right).$$ (70)

Many processes involving electron-transfers mechanism and molecular conductance rely on these especially characterized excited states.

### 6.1 Virial Theorem and Adiabatic Connection Theorem for Singlet-Triplet Splitting

Let us consider an excitation of a given system from a closed-shell ground state with an electronic configuration *φ*_*i*_ *φ*_*f*_. With the assumption of completely filled core with closed shell, this is made up of four Slater determinants: $|\varphi_{i}^{\alpha }\varphi_{f}^{\alpha }\rangle$ , $|\varphi_{i}^{\alpha }\varphi_{f}^{\beta }\rangle$ , $|\varphi_{i}^{\beta }\varphi_{f}^{\alpha }\rangle$ , and $|\varphi_{i}^{\beta }\varphi_{f}^{\beta }\rangle$ , where *α*, *β* denote up and down spins. Therefore, diagonalization of Hamiltonian matrix in the vicinity of the above four determinants gives the coupled excited states. The singlet state is given by $|\psi_{\text{S}}\rangle =\frac{1}{\sqrt{2}}\left\{|\varphi_{i}^{\alpha }\varphi_{f}^{\beta }\rangle -|\varphi_{i}^{\beta }\varphi_{f}^{\alpha }\rangle \right\}$ , and the three degenerate triplet states, on the contrary, is represented as follows: $\psi_{\text{T}}=|\varphi_{i}^{\alpha }\varphi_{f}^{\alpha }\rangle \;or\;\frac{1}{\sqrt{2}}\left\{|\varphi_{i}^{\alpha }\varphi_{f}^{\beta }\rangle +|\varphi_{i}^{\beta }\varphi_{f}^{\alpha }\rangle \right\}\;or\;|\varphi_{i}^{\beta }\varphi_{f}^{\beta }\rangle$ . The energies of respective singlets and triplets (denoted by “S” and “T” subscripts) have the form of

$$E_{\text{S}}=E^{\alpha \beta }+K_{if}$$ (71)

and

$$E_{\text{T}}=E^{\alpha \alpha }\;or\;E^{\alpha \beta }-K_{if}\;or\;E^{\beta \beta },$$ (72)

where $E^{\sigma_{1}\sigma_{2}}$ is the energy of a given determinant of form $|\varphi_{i}^{\sigma_{1}}\varphi_{f}^{\sigma_{2}}\rangle$ , (*σ*_1_, *σ*_2_) ∈ {*α*, *β*}, and *K*_*if*_ is the 2e^−^ integral (Coulomb self-energy of product of transition orbitals) defined as

$$K_{if}=\iint \frac{\varphi_{i}\left(r_{1}\right)\varphi_{f}\left(r_{1}\right)\varphi_{i}\left(r_{2}\right)\varphi_{f}\left(r_{2}\right)}{\left|r_{1}-r_{2}\right|}\;dr_{1}dr_{2}.$$ (73)

A combination of Eqs. 71 and 72 gives singlet and triplet excitation energies as follows:

$$E_{0S}=E_{0T}+2K_{if},$$ (74)

where *E*_0S_ = *E*_S_ − *E*_0_, *E*_0T_ = *E*_T_ − *E*_0_ and ground-state energy of the closed-shell system is denoted by *E*_0_. However, the problem in determining correlated STS energy makes Eq. 74 highly inaccurate for calculation of *E*_0S_. One way to deal with this is to use the well-known “adiabatic connection” theorem (Harris and Jones, 1974), which suggests that, in case of single-particle excitation, the single-triplet energy difference can be expressed in the form of

$$\Delta E_{\text{STS}}=\Delta E_{\text{STS}}^{0}+\Delta E_{\text{ST}}^{\text{corr}},\;\;\;\;\;\;\;\;\Delta E_{\text{STS}}=2K_{if}+\Delta E_{\text{ST}}^{\text{corr}}.$$ (75)

Here, $\Delta E_{\text{STS}}^{0}$ is the uncorrelated STS energy and $\Delta E_{\text{ST}}^{\text{corr}}$ represents the difference between singlet-triplet correlation energies.

Recently, a non-empirical formula (Becke, 2016) has been proposed to tackle the $\Delta E_{\text{ST}}^{\text{corr}}$ term. This is derived from the inter-electronic cusp condition and the effect it causes to electron correlation. Consequently, it can be written as

$$\Delta E_{\text{ST}}^{\text{corr}}=-0.4\;\int 4\;\varphi_{i}^{2}\left(r_{1}\right)\varphi_{j}^{2}\left(r_{1}\right)\;z_{\text{C}}^{2}\left[1-\frac{\ln\left(1+z_{\text{C}}\right)}{z_{\text{C}}}\right]dr_{1}.$$ (76)

In this prescription, the only unknown quantity is correlation length *z*_C_. This is nothing but the measure of spatial extent of electron correlation in configuration, *φ*_*i*_ *φ*_*f*_. In the limit of “strictly correlated electrons,” *z*_C_ can be expressed in terms of 2e^−^ integral, *K*_*if*_, as

$$0.4z_{\text{C}}^{2}\int \;4\;\varphi_{i}^{2}\left(r_{1}\right)\varphi_{j}^{2}\left(r_{1}\right)\;dr_{1}=2K_{if}.$$ (77)

Moreover, invocation of standard virial theorem to it (Becke, 2018a) allows one to write

$$\Delta E_{\text{ST}}^{\text{corr}}=-K_{if}.$$ (78)

This surprisingly leads to a further simplification, which reduces the relation to

$$\Delta E_{\text{STS}}=2K_{if}-K_{if}=K_{if}.$$ (79)

It is to be pointed out here that this is a completely non-empirical expression of correlated STS energy but a more simplified one, involving only the 2e^−^ integral.

Therefore, both routes prescribed in Eqs 76 and 79 are entirely non-empirical, implying Δ*E*_STS_ to be lower than 2*K*_*if*_. This leads to a formal definition of a molecule-independent re-scaling parameter *f* such that

$$\Delta E_{\text{STS}}=2fK_{if},\;0<f<1.$$ (80)

As long as the de-localization error (Perdew et al., 1982) is not a serious concern, determining this parameter in a semi-empirical way might offer overall good quality excitation energies. Optimization of *f* through a semi-empirical technique (Becke, 2016) gives the value as 0.486 when the results are fitted using the best approximated theoretical excitation energy data set of Silva-Junior et al. (2010). Surprisingly, this is close to the value (0.5) obtained from a consideration of the “virial theorem.”

Here, the central quantity, *K*_*if*_, is implemented in a manner considerably different from the original prescription (Becke, 2016). In place of the multi-center numerical integral procedure used in Becke and Dickson (1988), we employ an FCT scheme using an RS technique in CIK. As per the description in Ghosal et al. (2019), *K*_*if*_ can be recast as

$$K_{if}=\iint \frac{\varphi_{i}\left(r_{1}\right)\varphi_{f}\left(r_{1}\right)\varphi_{i}\left(r_{2}\right)\varphi_{f}\left(r_{2}\right)}{\left|r_{1}-r_{2}\right|}\;dr_{1}dr_{2}=\int \varphi_{i}\left(r_{1}\right)\varphi_{f}\left(r_{1}\right)v_{if}\left(r_{1}\right)dr_{1}.$$ (81)

Now, the tricky job is to evaluate the *v*_*if*_ integral, for which we employ the RS-FCT procedure once again. This can be further manipulated as

$$v_{if}\left(r_{1}\right)=\int \frac{\varphi_{i}\left(r_{2}\right)\varphi_{f}\left(r_{2}\right)}{\left|r_{1}-r_{2}\right|}dr_{2}=\int \frac{\varphi_{if}\left(r_{2}\right)}{\left|r_{1}-r_{2}\right|}dr_{2}=\varphi_{if}\left(r_{1}\right)⋆v^{\text{c}}\left(r_{1}\right).$$ (82)

The last expression involves the convolution integral, where *φ*_*if*_ indicates a simple multiplication of *i*th and *f*th MO from lowest triplet excited state, whereas *v* ^c^(**r**) signifies the CIK. Further simplification of this integral can be made using FCT as

$$v_{if}\left(r\right)=F^{-1}\left\{v^{\text{c}}\left(k\right)\varphi_{if}\left(k\right)\right\}\;where\;\varphi_{if}\left(k\right)=F\left\{\varphi_{if}\left(r\right)\right\}.$$ (83)

Here, *v* ^*c*^(**k**), *φ*_*if*_(**k**) represent Fourier integrals of Coulomb kernel and MOs. Other necessary quantities such as correlation length, *z*_C_, and difference in singlet-triplet correlation energy, are simply evaluated in the same essence, in real-space grid using pseudo KS orbitals *φ*_*i*_ and *φ*_*f*_.

### 6.2 Excitation Energies From Becke’s Exciton Model

The effectiveness of the above-mentioned approach is presented through an “SBKJC” type pseudopotential basis set, which is devoid of any diffused function. Furthermore, the lowest singlet and triplet excited states in this exploratory study correspond to the first single excitation for every molecule. Calculations are pursued with an optimized grid using a similar technique to that mentioned in previous sections. In this background, E_0*S*_ and E_0*T*_ (in eV), as well as correlated STS terms, are tabulated separately in Table 12, along with correlation length (*z*_C_), for B3LYP functional. A cross-section of molecules is presented from a larger set provided in Ghosal et al. (2021); current conclusions are drawn based on that set. Note that E_0*S*_ presented here is of both non-empirical and empirical nature: 1) PR_1_ uses Eq. 79, which is a semi-empirical approach with re-scaling parameter *f* = 0.486, 2) PR_2_ refers to a non-empirical model from “adiabatic connection” defined in Eq. 75, and 3) PR_3_ presents results obtained from Eq. 78 employing a non-empirical model from the virial theorem. In columns 6 and 8, corresponding TD-B3LYP energies (E_0*S*_, E_0*T*_) calculated from GAMESS (Schmidt et al., 1993) using the same functional and basis set are presented for side-by-side comparison. An analysis of E_0*S*_ suggests that PR_3_ improves results from PR_2_. The performance of PR_1_ is in close proximity to PR_3_. Therefore, out of three methods, PR_3_ proves to be the best estimate as those are quite competitive with TD-B3LYP. The next columns provide E_0*T*_ and Δ*E*_STS_ for an understanding of the contribution of these terms in calculating E_0*S*_. Though the overall agreement is satisfactory for both E_0*S*_ and E_0*T*_, the worst performance is observed for propene (relative to TD-B3LYP). A careful analysis suggests that the major source of inaccuracy in E_0*S*_ is the STS term, not E_0*T*_. This leads to the understanding that the success of our method depends on an accurate estimation of 2e^−^ integrals or, in other words, the accuracy of triplet states.

**TABLE 12:** E_0*S*_, E_0*T*_, and Δ*E*_STS_ (in eV) using B3LYP functional. For details, see Ghosal et al. (2021).

Molecule	State	*E*_0S_				*E*_0T_		Δ*E*_STS_		*z*_C_
		PR_1_	PR_2_	PR_3_	TD-B3LYP (Schmidt et al., 1993)	Reference (Roy et al., 2019)	TD-B3LYP	PR_3_	TD-B3LYP	
Ethylene	B_1*u*_(*π* → *π* ^⋆^)	7.78	7.63	7.87	8.09	4.47	4.03	3.40	4.06	2.97
Propene	A′(*π* → *π* ^⋆^)	7.18	7.05	7.26	7.81	4.44	4.03	2.82	3.78	2.99
1,3-Butadiene (E)	B(*π* → *π* ^⋆^)	5.63	5.42	5.70	6.02	3.26	2.71	2.44	3.31	3.29
1,3,5-Hexatriene (E)	B_*u*_(*π* → *π* ^⋆^)	4.38	4.14	4.44	4.79	2.42	1.85	2.02	2.94	3.53
1,3-Cyclo-pentadiene	A′(*π* → *π* ^⋆^)	5.12	5.03	5.17	5.28	3.21	2.70	1.96	2.58	2.98
Thiophene	B_2_(*π* → *π* ^⋆^)	5.61	5.31	5.66	6.02	3.88	3.47	1.78	2.55	3.99
Acetaldehyde	A′′(*n* → *π* ^⋆^)	4.67	4.75	4.68	5.07	4.39	4.44	0.29	0.24	1.44

In order to validate the usefulness of the proposed method and enlarge the scope of applicability, some larger molecular systems are approached in Table 13. Thus, *E*_0S_, *E*_0T_ for some representative organic chromophores and linear acenes (Ghosal et al., 2021) are presented. Geometries of these systems are taken from Silva-Junior et al. (2010), whereas the same for linear acenes are obtained from GAMESS calculation using the B3LYP functional and CC-pVDZ basis. In addition to B3LYP, LC-BLYP from the family of RSH functionals is also invoked. All the results henceforth refer to the PR_3_ calculation. Apparently, the performance of B3LYP is much more consistent than that of LC-BLYP; the former shows an overestimation in excitation energies. Also, instead of a dramatic one, only a subtle betterment of results is observed using LC-BLYP. The effect of full HF exchange at LR has no dramatic effect on excitation energies, although it enhances the behavior of frontier orbitals used in *K*_*if*_ computations. This discordance has occurred as *γ* is assumed to be independent of system size. It is possible to achieve a greater level of performance by treating *γ* as a system-dependent parameter (functional of *ρ*) estimated from the first principles (Baer et al., 2010). It is believed that an optimally tuned (in the spirit of the size-dependency principle) *γ* will outperform the conventional hybrid and RSH functionals in terms of results. An analogous qualitative trend is also depicted by linear acenes.

**TABLE 13:** Excitation energies of organic chromophores and linear acenes from “virial theorem.” These are taken from Ghosal et al. (2021).

Orgnaic chromophores						
Molecule	State	*E*_0T_ (eV)		*E*_0S_ (PR_3_) (eV)		Lit.^a^ (eV)
		B3LYP	LC-BLYP	B3LYP	LC-BLYP	
Cyclopropene	B_2_(*π* → *π* ^⋆^)	4.03	4.05	7.04	7.07	7.01
Norbornadiene	A_2_(*π* → *π* ^⋆^)	4.62	4.23	5.77	5.45	4.91
Naphthalene	B_2*u*_(*π* → *π* ^⋆^)	3.22	3.53	4.74	5.20	4.64
Pyridazine	B_3*u*_(*n* → *π* ^⋆^)	2.76	2.78	3.35	3.34	3.57
Acetamide	A′′(*n* → *π* ^⋆^)	5.20	5.15	5.45	5.37	5.46
**Linear acenes**						
**Number of rings**						
2	—	3.24	3.56	4.75	5.22	4.65
3	—	2.22	2.46	3.71	4.43	3.58
4	—	1.55	1.81	2.85	3.41	2.75
5	—	1.08	1.32	2.30	2.96	2.22
6	—	0.75	0.98	1.89	2.50	1.82

a This corresponds to *E*_0S_ from Becke (2018a).

#### 6.2.1 Basis-Set Dependence

This section produces optical gaps generated from *n* → π* and *π* → π* transitions in selected molecules in Table 14. In order to probe the dependence on the basis set, singlet excitation energies are reported employing two basis sets, namely, 6-311G (B1) and 6-311 + +G* (B2), both with B3LYP functional. The corresponding geometries are taken from supplementary materials of Silva-Junior et al. (2010). All calculations are done with all-electron orbitals. The energies E_0_ and E_*T*_ are calculated using the GAMESS program package. The triplet calculations correspond to restricted open-shell. The K_*if*_ integrals are evaluated numerically with our InDFT program (Roy et al., 2019), taking orbitals from GAMESS. The results are compared with the “theoretical best estimate” TBE-2 (Silva-Junior et al., 2010) benchmark values tabulated in column 3 and are comparable. A detailed analysis in terms of MAE and ME values for a larger set is available in Roy et al. (2021), of which Table 15 is a subset. Now, similar to our previous conclusion, these results are also comparable with TD-B3LYP energies; in fact, the ones with B2 are in better agreement (Roy et al., 2021). This reflects the basis set dependency of these quantities. It is also delineated by Roy et al. (2021) that TD-B3LYP significantly underestimates the excitation energies from the current procedure.

**TABLE 14:** E_0*S*_ (in eV) in organic dyes, using B3LYP functional. See Roy et al. (2021) for details.

Molecule	State	TBE-2 (Silva-Junior et al., 2010)	TD-B3LYP (B1)	PR (B1)	TD-B3LYP (B2)	PR (B2)
Ethene	B_1*u*_(*π* → *π* ^⋆^)	7.80	7.99	8.07	7.41	7.71
*E*-Butadiene	B_*u*_(*π* → *π* ^⋆^)	6.18	5.98	6.31	5.58	6.06
Cyclopentadiene	B_2_(*π* → *π* ^⋆^)	5.55	5.20	5.58	4.96	5.38
Norbornadiene	A_2_(*π* → *π* ^⋆^)	5.37	5.03	6.10	4.71	5.62
Naphthalene	B_2*u*_(*π* → *π* ^⋆^)	4.82	4.50	4.79	4.31	4.63
Imidazole	A′(*π* → *π* ^⋆^)	6.25	6.15	7.09	5.11	4.61
Pyridine	B_1_(*n* → *π* ^⋆^)	4.59	3.84	3.71	3.94	3.82
Pyrazine	B_3*u*_(*n* → *π* ^⋆^)	4.13	3.84	3.71	3.94	3.82
*p*-benzoquinone	B_1*g*_(*n* → *π* ^⋆^)	2.74	2.38	2.52	2.44	2.55
Uracil	A′′(*n* → *π* ^⋆^)	5.00	4.60	4.52	5.13	5.56

#### 6.2.2 Dependence on XC Functional

**FIGURE 3:**
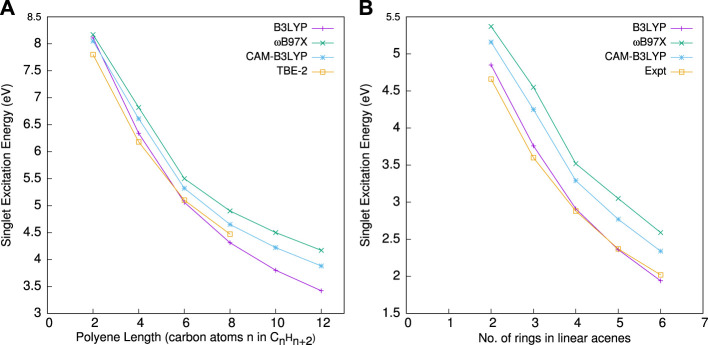
E_0*S*_ with different functionals against **(A)** polyene length and **(B)** the number of rings. Both panels employ a B1 basis. More details are given by Roy et al. (2021).

Next, in Figure 3, we investigate the functional reliance of the present approach. In this regard, in left and right panels, E_0*S*_ for polyenes and linear acenes with three different functionals, namely, B3LYP, *ω*B97X, and CAM-B3LYP, are plotted with respect to polyene length and the number of rings. These three functionals have a variable amount of EEX contribution throughout the inter-electronic distance. This study aims to demonstrate the role of EEX in determining the optical gap in systems with reduced HOMO-LUMO gap. When the chain length (or rings) increases, the gap squeezes. As one moves from panels (a) to (b), the optical gap exhibits a similar pattern. This feature is well reproduced by all three functionals, but when compared to TBE-2 (Silva-Junior et al., 2010), B3LYP outperforms all of them. Specifically, *ω*B97X deviates most from reference, followed by CAM-B3LYP. The impact of EEX in the LR region is less important; rather, an appropriate balance between EEX and correlation is required throughout the inter-electronic distances. This is consistent with the fact that, unlike Rydberg and CT excitations, the optical gap has no LR feature. According to Becke (2018b), the optimal contribution of EEX is around 21%; thus, the success of B3LYP over CAM-B3LYP and *ω*B97X is easily understandable. When comparing CAM-B3LYP with *ω*B97X, the former wins as EEX’s contribution in SR is still minor when compared to *ω*B97X. However, the system-independent value of the RS parameter in *ω*B97X and CAM-B3LYP fails to induce the size dependency of the optical gap problem, implying that this size-dependency component is necessary in the parameter. Overall, the present method shows sensitivity toward EEX contributions in SR (rather LR).

#### 6.2.3 Optical Gaps in Organic Chromophores

Now, we explore a few applications pertaining to the photoluminescence effects in organic chromophores. This requires a detailed account of low-lying excited states. In this regard, the HOMO-LUMO gap (E_*L*−*H*_) and E_0*S*_ for a few representative linear and nonlinear poly-cyclic aromatic hydrocarbons (PAH) and organic dyes (comparatively difficult systems) from Roy et al. (2021) are tabulated in Table 15. Geometries for the organic dyes are supplied by Kowalczyk et al. (2011). These correspond to B3LYP/cc-PVTZ calculations. Along with the calculated excitation energies, experimental and TD-B3LYP results are also presented here. The consistency in overall performance is quite encouraging. A careful analysis (Roy et al., 2021) shows that PR is comparable with TD-B3LYP for both PAHs and organic dyes. While excitation energies calculated with TD-B3LYP shows systematic underestimation, the present scheme, in contrast, shows an overestimation consistently, which follows Becke (2018a). For organic dyes, while the efficiency of TD-B3LYP is superior to the current approach, the inaccuracy is more consistent in the latter. Note that the energies obtained for organic dyes have shown sensitivity toward the chosen basis set.

**TABLE 15:** HOMO-LUMO gap (*E*_L-H_), HOMO-LUMO singlet excitation energy in organic chromophores. PR ≡ present result. Details are available in Roy et al. (2021).

Linear acenes				
Rings	*E*_L-H_ (eV)	Expt. (eV)^a^	TD-B3LYP (eV)	PR (eV)
2	4.91	4.66	4.57	4.85
3	3.66	3.60	3.37	3.76
4	2.84	2.88	2.57	2.91
5	2.26	2.37	2.02	2.36
6	1.84	2.02	1.60	1.94
**Non-linear PAHs**				
Phenanthrene	4.84	4.35	4.40	4.76
Benzo[e]pyrene	4.10	3.84	3.87	4.25
Dibenz[a,c]anthracene	3.10	3.95	3.61	4.07
anthanthrene	2.94	2.97	2.92	3.34
**Organic dyes**				
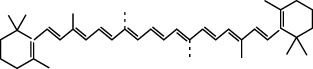	2.22	2.50	2.23	2.44
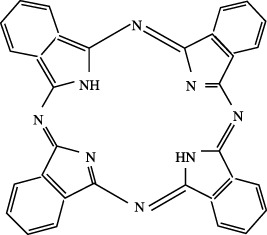	2.18	1.82	2.11	2.52
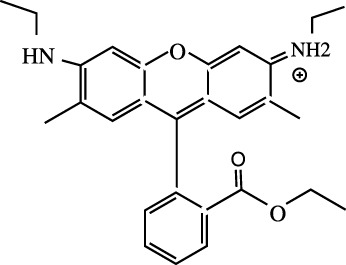	2.94	2.26	2.85	2.95
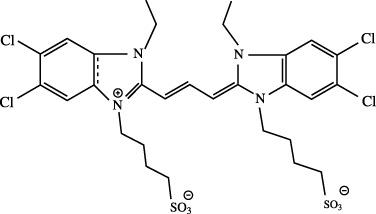	2.96	2.11	2.71	2.78

a Experimental values are obtained from Grimme and Parac (2003) for linear acenes, Parac and Grimme (2003) for non-linear PAHs, and Kowalczyk et al. (2011) for organic dyes.

#### 6.2.4 Charge-Transfer Excitation Within a Hybrid (G)KS Framework

The working equation on this occasion is Eq. 79. The respective single-point energies (E_0_ and E_*T*_) are obtained from conventional DFT calculations using Gaussian 09 package (Frisch et al., 2016). Full calculations are performed with the cc-PVTZ basis set employing BLYP, B3LYP, and LC-BLYP functionals. Similar to the previous case, K_*if*_ integrals are accomplished using InDFT (Roy et al., 2019). Two different kinds of CT complexes are chosen, which are relatively hard to deal with numerically. First, we consider weakly bound CT complexes (first four molecules of CT-A of Table 16). They are bounded with non-covalent bonds of length in the range of 3.2–3.6 Å, making them quite attractive and challenging. A set of intra-molecular CT complexes is also studied (last three of CT-A). Finally, we look at some harder organic compounds (presented in column CT-B) that display thermally activated delayed fluorescence (TADF). The geometry of these molecules is sourced from the supplementary materials of Hait et al. (2016). In Table 16, excitation energies, PR_*n*_, calculated from the current scheme and TD_*n*_ referring to the same with TDDFT, are presented. Here, subscript “*n*” symbolizes three XC functionals; *n* = 1, 2, 3 stands for BLYP, B3LYP, and LC-BLYP. For comparison, literature/experimental results are also provided side by side. Now, energies from restricted open shell or unrestricted calculations are reported as per convergence; we have taken this liberty as the difference between these two calculations is not significant enough to bring any perceptible change in excitation energy (Roy et al., 2022). As we shift from BLYP to LC-BLYP *via* B3LYP for both PR and TD, a general trend of increment in excitation energy is noticed in both CT complexes. An in-depth examination indicates that, for molecules in CT-A, the impact of K_*if*_ gets dominated by E_0*T*_ for all the XC functionals considered here. Thus, in the framework, to a large extent, E_0*T*_ determines the observed trend in E_0*S*_. However, for systems in CT-B, there is no way to determine *a priori* which will be a dominant factor between E_0*T*_ and K_*if*_ for a given case. Therefore, for systems with E_0*T*_ dominance over K_*if*_, B3LYP performs better than RSH functionals, but when their contributions are comparable, the mutual cancellation of these determines the excitation energy.

**TABLE 16:** E_0S_ from the present result (PR) and TDDFT (TD) in some CT complexes. NC implies “not converged.” More details are available in Roy et al. (2022).

Weakly bound CT complex								TADF exhibiting CT complex							
(CT-A)								(CT-B)							
System	PR_1_	PR_2_	PR_3_	TD_1_	TD_2_	TD_3_	Reference	System	PR_1_	PR_2_	PR_3_	TD_1_	TD_2_	TD_3_	Expt. (Hait et al., 2016)
Hexamethylbenzene-TCNE^a^	NC	1.92	1.92	0.79	1.09	2.74	2.36 (Risthaus et al., 2014)	2CzPN	2.87	3.06	3.42	2.26	2.85	4.29	3.19
Diphenylene-TCNE^b^	1.82	3.06	1.96	0.72	0.82	2.63	2.28 (Risthaus et al., 2014)	4CzPN	NC	2.64	3.23	1.84	2.48	4.10	2.82
Hexamethylbenzene-chloranil^a^	NC	2.09	2.54	0.84	1.30	3.28	2.87 (Risthaus et al., 2014)	4CzTPN	2.06	2.41	3.35	1.66	2.24	3.75	2.61
Diphenylene-chloranil^b^	1.87	2.29	2.48	1.24	1.49	3.89	2.81 (Risthaus et al., 2014)	ACRFLCN	NC	2.87	4.48	1.82	2.52	4.75	3.05
DCS	2.87	3.16	3.98	2.69	3.07	3.76	3.59 (Brémond et al., 2021)	PXZ-TAZ	3.01	3.46	4.32	1.91	2.73	4.60	3.33
DANS^a^	2.60	2.96	3.84	2.14	2.65	3.64	3.45 (Brémond et al., 2021)	DPA-DPS	3.25	3.94	5.60	2.81	3.42	4.35	3.53
Coumarin-152	3.81	3.92	4.42	2.96	3.39	4.06	3.72 (Brémond et al., 2021)	PXZ-OXD	NC	3.14	4.39	1.50	2.33	4.33	3.18

a RO-BLYP calculation did not converge in this particular case.

b GAMESS software (Schmidt et al., 1993) was employed as RO-calculation did not converge in Gaussian09.

**FIGURE 4:**
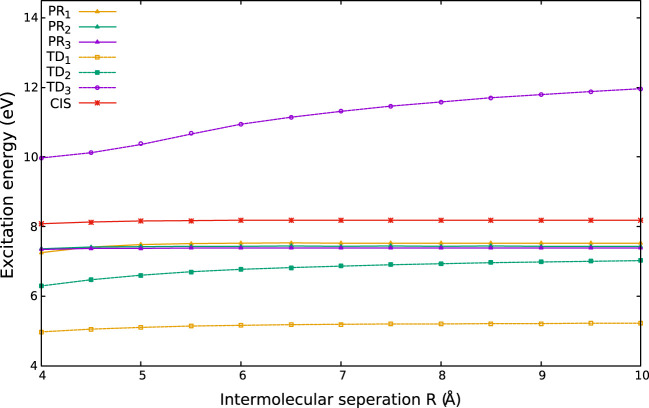
E_0S_ for C_2_ H_4_ − C_2_ F_4_ *versus* R. PR_1_, PR_2_, and PR_3_ denote the present result with BLYP, B3LYP, and LC-BLYP functional, and TD_1_, TD_2_, and TD_3_ represent the same within TDDFT. Adapted from Roy et al. (2022).

Finally, we proceed to investigate the asymptotic limit of CT excitation in some weakly interacting systems, characterized by *R* ^−1^ energy decay (*R* is inter-molecular separation). This study also intends to exploit the perseverance of the present scheme throughout *R*. With this in mind, in Figure 4, the excitation energy of an inter-molecular dimer C_2_H_4_ − C_2_F_4_ is depicted as a function of *R* for functionals that have already been mentioned. Results are compared with configuration interaction singles (CIS) (Foresman et al., 1992) which may be a benchmark. For the entire region, all the PR_*n*_(*n* = 1–3) energies are in admissible accordance with CIS, without any substantial difference between them. However, this is not true for TD_*n*_(*n* = 1–3) results. They remain distinctly separated. TD_3_ offers overestimation from CIS, whereas the other two record underestimation. Note that the B3LYP result (Feng et al., 2018) within the “virial theorem” model slightly overestimates CIS for the entire region of *R*, whereas PR_2_ energies are underestimated. This lean disparity perhaps is introduced from two separate numerical strategies used for the calculation of K_*if*_. In contrast to the present approach, TDDFT appears to be more sensitive to *R* and shows CT breakdown. Surprisingly, TD_3_ appears to provide the poorest result. This is mainly due to the system independence of *γ*. The invocation of a size-dependent *γ* possibly can partly alleviate this problem.

## 7 Future and Outlook

We have exhibited the legitimacy and viability of the Gaussian-based LCAO-MO approach to DFT involving CCG in the context of atomic and molecular properties. A wide variety of atoms and molecules are used as test beds to examine the efficacy of CCG in the context of ***μ***, ***α***, ***β*** with an optimized FF procedure. Comparison with existing theoretical and experimental data vouch for its suitability and effectiveness. The feasibility and practicability of a direct NR approach for coupling accurate exchange energy density, which is a key component of hyper-type of functionals, energy, and matrix in real-space CCG, is discussed. This was done for a variety of atoms and molecules, with properties including total energy and orbital energies. These were also shown for B3LYP, PBE0, and BHLYP hybrid functionals. The effectiveness of this strategy is reliant on the precise estimation of ESP integral, which in turn, depends on the optimization of the RS parameter in CIK. The scaling suggests that this approach could be very useful in massive large-scale DFT calculations incorporating orbital-dependent functionals. Most importantly, within the CCG, the NR scheme appeared to be more proficient than the alternative SNR-OS technique. Carrying forward the success of this approach, in the following segment, the suitability and performance of a self-consistent systematic optimization procedure for OT-RSH functionals are presented. Their performance was assessed by probing properties such as frontier orbital energies, fractional occupation of electron on HOMO, and fundamental gap. For finite systems, the predominance of OT-RSH functionals is observed over the respective RSH functionals. The success of this method relies on the precise estimation of *γ*_OT_ based on the size-dependency principle.

We then move to the realm of excited states (within a time-independent scheme) and detail the practicability and convenience of a simple yet accurate TIKS-DFT approach, to calculate single excitation energies in a realistic manner. This was tested for a host of linear acenes having *π* network, organic chromophores, linear acenes, and charge-transfer complexes. The derived results from the virial theorem are in appreciable accordance with reference results for all species. This simple scheme has been exhibited as a feasible choice for predicting optical gaps in organic chromophores. The above-mentioned outcome of the CCG method encourages us to use more effective core potential, more elaborate and sophisticated basis sets, and superior quality density functionals (e.g., RSH, hyper, and local hybrid XC functionals) to study different physicochemical aspects of many-electron systems. It might likewise be alluring to inspect its performance in a variety of exciting configurations apart from the lowest excited state. This approach could be highly beneficial in real-time dynamical approach, especially laser-molecules interactions in the intense domain within the TDDFT framework. In this pursuit, early results have very recently got published in Ghosal and Roy (2022b). In this article, the application of an intense laser field on electron dynamics of H_2_ and N_2_ molecules has been performed using real-time TDDFT. Moreover, other than single-point calculations, it would also be interesting to assess the merit and suitability of the current method for geometry optimization of molecules in CCG. A significant concern would be to reduce the computation cost by invoking a linear scaling technique. Some of these works are presently being scrutinized.
